# Theory-driven design of cadmium mineralizing layered double hydroxides for environmental remediation†

**DOI:** 10.1039/d4sc02860k

**Published:** 2024-07-19

**Authors:** Zixian Li, Nuo Xu, Jing Ren, Haigang Hao, Rui Gao, Xianggui Kong, Hong Yan, Xiao Hua, Yung-Kang Peng, Shulan Ma, Dermot O'Hare, Yufei Zhao

**Affiliations:** a State Key Laboratory of Chemical Resource Engineering, Beijing University of Chemical Technology Beijing 100029 P. R. China zhaoyufei@mail.buct.edu.cn; b Quzhou Institute for Innovation in Resource Chemical Engineering Quzhou 324000 Zhejiang P. R. China; c College of Chemistry and Chemical Engineering, Inner Mongolia University 010021 Hohhot Inner Mongolia P. R. China; d Department of Chemistry, Lancaster University Lancaster LA1 4YB UK; e Department of Chemistry, City University of Hong Kong Hong Kong Hong Kong SAR 999077 P. R. China; f Beijing Key Laboratory of Energy Conversion and Storage Materials and College of Chemistry, Beijing Normal University Beijing 100875 P. R. China; g Chemistry Research Laboratory, Department of Chemistry, University of Oxford Mansfield Road Oxford OX1 3TA UK

## Abstract

The environmental concern posed by toxic heavy metal pollution in soil and water has grown. Ca-based layered double hydroxides (LDHs) have shown exceptional efficacy in eliminating heavy metal cations through the formation of super-stable mineralization structures (SSMS). Nevertheless, it is still unclear how the intricate coordination environment of Ca^2+^ in Ca-based LDH materials affects the mineralization performance, which hinders the development and application of Ca-based LDH materials as efficient mineralizers. Herein, we discover that, in comparison to a standard LDH, the mineralization efficiency for Cd^2+^ ions may be significantly enhanced in the pentacoordinated structure of defect-containing Ca-5-LDH utilizing both density functional theory (DFT) and *ab initio* molecular dynamics (AIMD) simulations. Furthermore, the calcination-reconstruction technique can be utilized to successfully produce pentacoordinated Ca-5-LDH. Subsequent investigations verified that Ca-5-LDH exhibited double the mineralization performance (421.5 mg g^−1^) in comparison to the corresponding pristine seven coordinated Ca-7_OH/H_2_O_-LDH (191.2 mg g^−1^). The coordination-relative mineralization mechanism of Ca-based LDH was confirmed by both theoretical calculations and experimental results. The understanding of LDH materials and their possible use in environmental remediation are advanced by this research.

## Abstract 2

The environmental concern posed by toxic heavy metal pollution in soil and water has grown.

## Graphical abstract

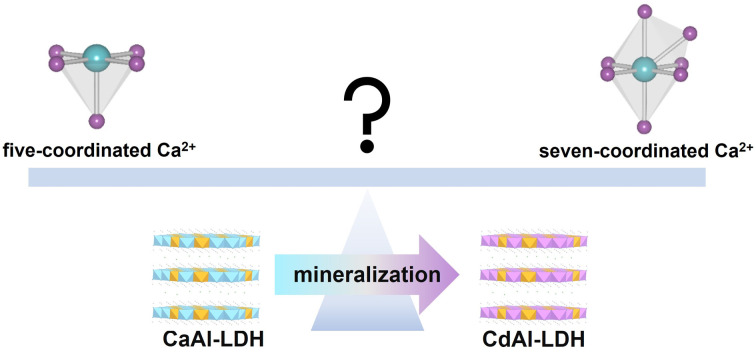

## Introduction

Heavy metals including Cd, Ni, Pb, and Cu are being released into our environment at an alarming rate due to the acceleration of global industrialization.^1,2^ The accumulation of heavy metal ions in organisms presents challenges in terms of metabolism and poses a severe threat to both the ecosystem and human health.^3,4^ Recently, a range of remediation methods, such as chemical precipitation,^5^ adsorption,^6^ ion exchange,^7^ and electrochemical methods,^8^ have been explored to remove such severe pollution.^9^ Due to its simple implementation and quick adsorption properties, the *in situ* immobilization method—which uses appropriate mineralizers to lower heavy metal concentrations by adsorption and/or precipitation—has attracted the most attention among these techniques.^10^ Nevertheless, the current capacity and stability of the available mineralizers in “fixing” heavy metals are still deemed unsatisfactory. Consequently, there has been a heightened endeavor to explore smart mineralizers that possess a superior removal capacity and stability.^11^

Anionic clay minerals known as layered double hydroxides (LDHs)^12^ are made up of layers of divalent and trivalent metal cations with six-octahedral coordination and negatively charged anions acting as interlayer guests.^13,14^ According to recent studies, LDHs have a powerful potential to mineralize heavy metal contaminants by creating super-stable mineralization structures (SSMS).^15,16^ Kong *et al.* used CaAl-LDH as the mineralizer to selectively remove Cd^2+^ ions from water^17^ and our group previously reported CaFe-LDH as an effective mineralizer to remove Ni^2+^ ions from wastewater^18^ and CaAl-LDH as a rapid stabilizer to mineralize Cu^2+^, Zn^2+^, Co^2+^, and Ni^2+^ ions.^19^ Even though the majority of previously published research concentrated on the removal capabilities of Ca-based LDHs, the relationship between the Ca coordination environment and performance has not been fully elucidated. A large amount of research has focused on understanding the structure of amorphous calcium carbonate^20^ and key outstanding problems include rationalizing its metastable state.^21^ An appropriate approach is to conduct complex structural evolution simulations.^22,23^ Similar studies on the structural evolution of calcium in solid minerals are quite rare,^24^ while previous work has lacked exploration of performance related relationships after structural recognition. Considering that Ca has a substantially higher atomic radius (1 Å) compared to the more typical divalent ions present in LDHs such as Mg (0.72 Å).^25^ the coordination environments of Ca ions in LDHs exhibit considerable diversity, frequently having coordination numbers of seven or more.^26^ Such a wide range of coordination environments can significantly influence the catalytic performance, such as in aldol condensations.^27–29^ Currently, there is a lack of elucidation of a relationship between the coordination environment of Ca cations in the LDH layers and the mineralization performance. Hence, it is imperative to investigate the structural evolution of calcium in solid-state LDHs and how it affects the mineralization properties, to explain this complex chemical kinetic behavior at the atomic level.

**Scheme 1:**
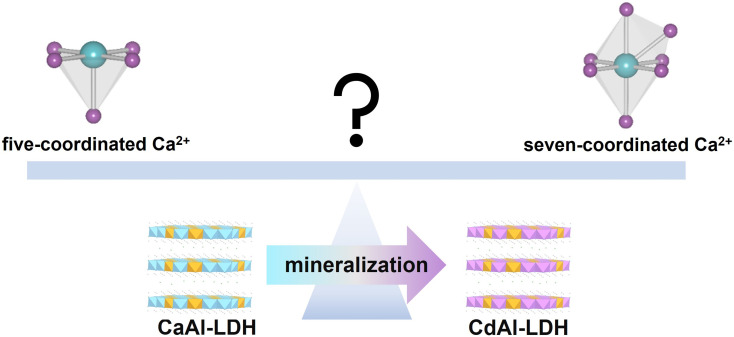
The effect of the Ca^2+^ coordination environment on the mineralisation of Cd^2+^ ions by LDHs.

Density functional theory (DFT) and *ab initio* molecular dynamics (AIMD) were used to determine the structure of Ca_2_Al_1_-LDH. In the pristine Ca_2_Al_1_-LDH structure, Ca ions are six-coordinated by oxygen ions (Ca-6-LDH). The Ca coordination number (CaAl-5-LDH) will decrease with the formation of oxygen vacancies, while additional coordination with molecules in the solution will increase the Ca coordination number (Ca-7_x_-LDH, x stands for the Cl^−^, OH^−^ or H_2_O). This work aims to explore the effect of Ca^2+^ ion coordination on the mineralization performance (Scheme 1). Our theoretical results indicate that a defective LDH (Ca-5-LDH) will promote the mineralization process towards the removal of Cd^2+^ ions due to lower steric hindrance and favorable coordination with water molecules, and subsequent experimental results confirmed this conclusion. This work innovatively utilizes theoretical DFT and AIMD methods to quantitatively analyze the coordination environment of Ca ions on LDH surfaces, as well as the thermodynamics and kinetics of Ca-solvent coordination, to provide theoretical guidance for subsequent thermodynamic calculations and dynamic simulations in the study of environmental remediation materials.

## Results and discussion

### Structure confirmation

Ca_2_Al_1_-Cl-LDH was chosen for theoretical modeling (Fig. S1†) as chloride is a common anion that could be utilized when using Cl-containing salts as the raw source.^30^ Within the LDH layers, six hydroxyl groups surround each divalent or trivalent cation, forming a hexacoordinated octahedral structure as shown in Fig. S1b† (Ca-6-LDH).^30^ Furthermore, it is worth noting that hydroxyl defects are commonly present in the layers of LDHs.^31^ These defects have been found to significantly enhance the catalytic and mineralization capabilities of LDHs.^32^ The presence of hydroxyl defects leads to a pentacoordinated structure (Ca-5-LDH) as illustrated in Fig. S1a.† Previous literature has reported that due to the large radius of the Ca^2+^ ions, they form additional coordination with either the intercalated solvent or the interlayer anions.^33^ Considering the composition of the alkaline environment, it is possible for Cl^−^, OH^−^, or H_2_O to act as the seventh coordination to Ca^2+^ within the LDH structure, Ca-7_Cl_-LDH (Fig. S1c†), Ca-7_OH_-LDH (Fig. S1d†) and Ca-7_H_2_O_-LDH (Fig. S1e†), respectively.

**Fig. 1:**
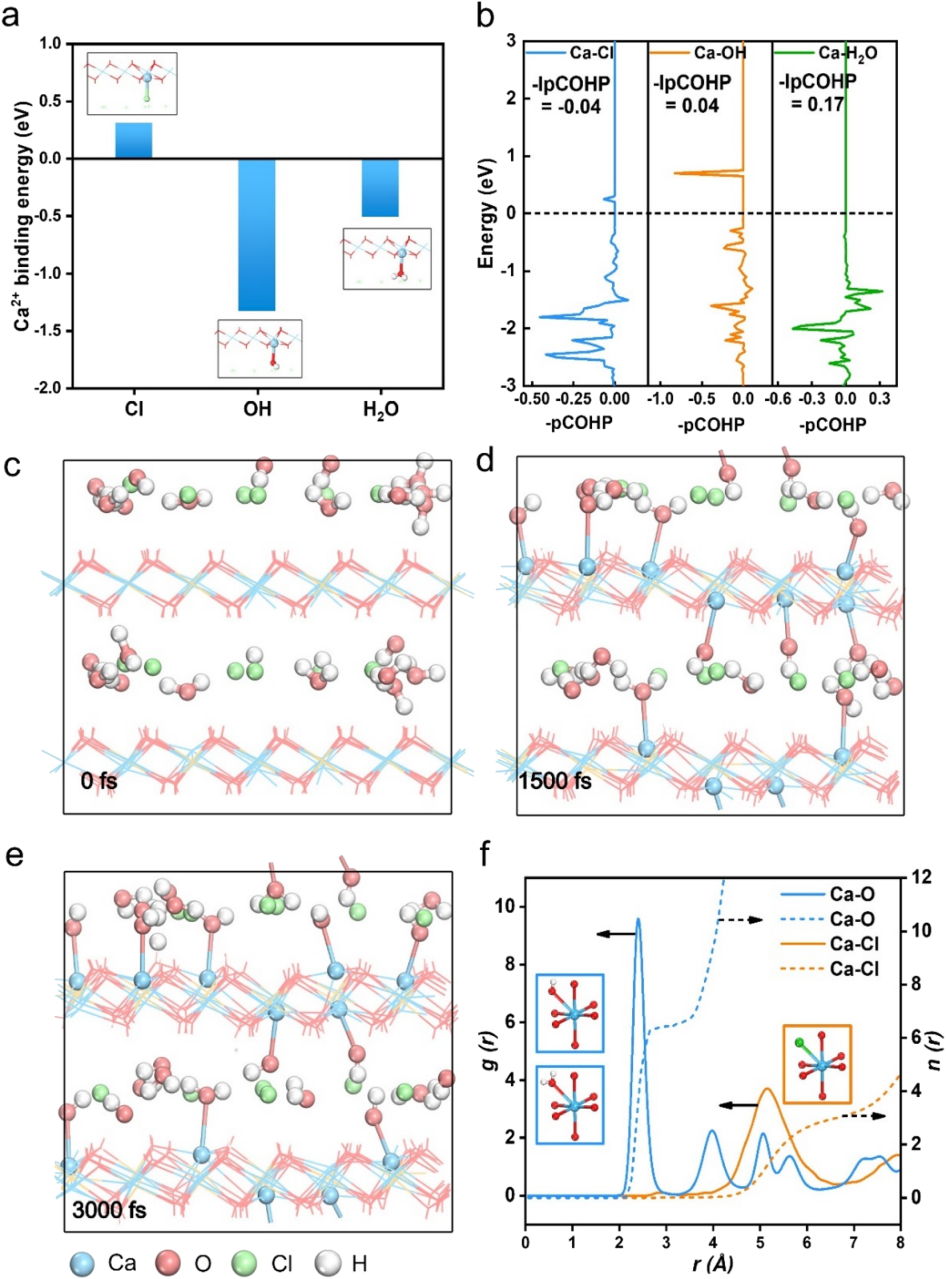
(a) Comparison of binding energies between Ca^2+^ and the seventh ligand (Ca-Cl/Ca-OH/Ca-H_2_O bond in Ca-7_Cl_-LDH/Ca-7_OH_-LDH/Ca-7_H2O_-LDH, respectively); (b) COHP analysis of additional coordination bonds, Ca-Cl/Ca-OH/Ca-H_2_O bonds in the Ca-7_Cl_-LDH/Ca-7_OH_-LDH/Ca-7_H2O_-LDH structure, respectively. IpCOHP is the integral of COHP, the more positive the value, the stronger the bond. The configurations of the AIMD process of the Ca-6-LDH structure at (c) 0 fs; (d) 1500 fs and (e) 3000 fs; (f) RDF analysis of the AIMD process.

In order to determine the final stable structure of Ca-7_x_-LDHs, we initially calculated the energy for three ligands (Cl^−^, OH^−^ and H_2_O) combined with Ca^2+^ (Fig. 1a). It can be seen that the binding energy of the OH^−^ and H_2_O ligands to Ca^2+^ is negative (−1.33 eV for the Ca-OH bond and −0.51 eV for the Ca-H_2_O bond), and that of Cl^−^ is much positive (0.31 eV for the Ca-Cl bond), which suggested that the combination of the OH^−^ and H_2_O ligands with Ca^2+^ is thermodynamically spontaneous, while Cl^−^ ligands cannot directly form a bond with Ca^2+^. We further analyzed the structural parameters of the Ca^2+^ and the surrounding ligands when the seventh coordination structure was formed (Table S1,† the corresponding structure is displayed in Fig. S2†), and the results show that when Ca^2+^ forms a bond with the seventh ligand, the bond length with the hydroxyl on the laminate still lies in the Ca–O bonding range (∼2.5 Å).^34^ This proves that the formation of the seventh coordination did not destroy the original hexacoordinated octahedral structure of the laminate. Crystal orbital Hamilton population (COHP) analysis showed the contribution of bonding and antibonding states to the energy of the band structure and was commonly used to compare the bond strength.^35^ The COHP analysis of the bond between Ca^2+^ and the seventh ligand (Cl^−^, OH^−^ and H_2_O) is shown in Fig. 1b. IpCOHP was used to quantitatively investigate the bond strength, and the values of Ca-OH and Ca-H_2_O bonds are 0.04 and 0.17, respectively, and the value of the Ca–Cl bond is −0.04, which proves that Ca^2+^ tends to form stronger bonds with OH^−^ and H_2_O, and the orbital repulsion between Ca^2+^ and Cl^−^ is too strong to form a bond, which is in accordance with Fig. 1a.

In order to further verify the results that the O atom of OH^−^ and H_2_O is more favorable to bond with Ca^2+^, we used the AIMD method to simulate the dynamic behavior of the Ca-6-LDH structure in a solution environment. 3000 fs kinetic simulation calculations were performed, and energy and temperature changes are shown in Fig. S3.† The energy and temperature changes prove that our dynamic simulation system has reached equilibrium. Fig. 1c–e are the structural snapshots at 0 fs, 1500 fs, and 3000 fs of the kinetic simulation, respectively. Configurations show that Ca^2+^ forms an extra bond with O atoms (both from OH^−^ and H_2_O) in the solution. The radial distribution function (RDF) analysis could be used as an efficient tool to gather statistics of the distribution around the target atoms and therefore to investigate the coordination situation.^36^ The RDF analysis of the kinetic process is shown in Fig. 1f. The coordination number is about 6.5 when the Ca–O bond length is in the range of about 2.5 Å, and a larger coordination number indicates that Ca^2+^ forms a new coordination with O (from OH^−^ and/or H_2_O) in the interlayer region. The distance between Ca and Cl^−^ is mostly concentrated between 5 and 6 Å, which is too far to be in the bonding range, and no obvious coordination structure is observed. DFT calculation combined with AIMD simulation confirmed the possibility of Ca^2+^ within the LDH structure forming a seven-coordinate structure with OH^−^ and H_2_O in the interlayer region, while the stability of the constructed pentacoordinated structure in Ca-5-LDH is also explored below.

### Isomorphic substitution of Cd^2+^

After determining the structure of the multi-coordinate environment of Ca^2+^ ions within the LDH structure, the energy changes upon isomorphic substitution of Ca^2+^ by Cd^2+^ ions in Ca-5-LDH, Ca-6-LDH, Ca-7_OH_-LDH and Ca-7_H_2_O_-LDH during the mineralization process have been investigated. According to prior literature studies,^19,37,38^ dissolution of Ca^2+^ ions, stabilization of Cd^2+^ ions, and the formation of CdAl-LDH are proposed for the mineralization process. This is reasonable based on the similar ionic radius of Ca^2+^ (1.00 Å) and Cd^2+^ (0.95 Å). From a theoretical perspective, the change in free energy upon gradually replacing Ca^2+^ ions in CaAl-LDH with Cd^2+^ ions may be used to probe the isomorphic substitution process.

The chemical reaction process is described as follows:Ca_12_Al_6_(OH)_36_(Cl)_6_ + *n*Cd^2+^ → Cd_*n*_Ca_12−*n*_Al_6_(OH)_36_(Cl)_6_ + *n*Ca^2+^where *n* = 12 would correspond to a complete isomorphic substitution process.

**Fig. 2:**
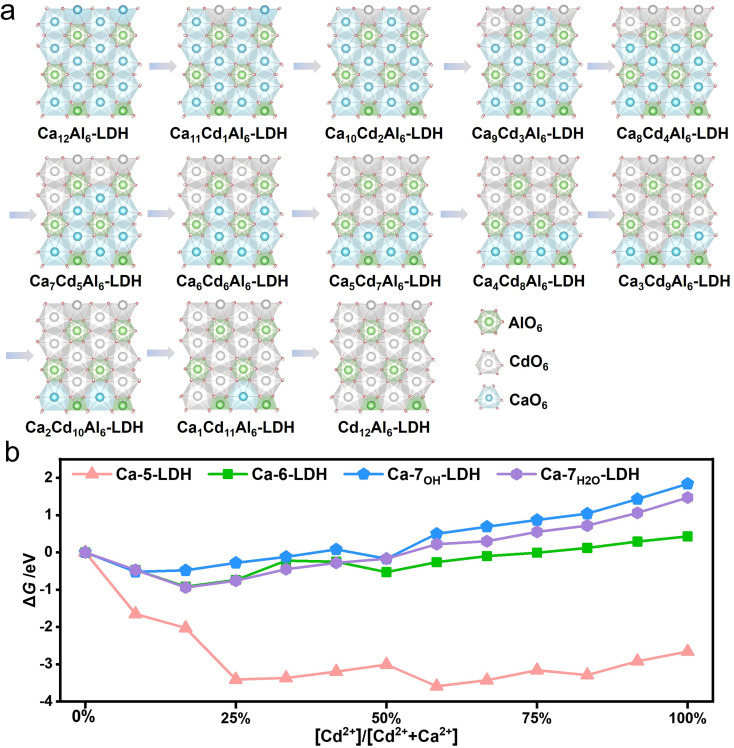
(a) Optimized geometries of the corresponding reaction intermediates in the isomorphic substitution of Cd^2+^ by Ca-6-LDH; (b) relationship between the Gibbs free energy and the ratio of [Cd^2+^] to [Cd^2+^ + Ca^2+^] in the isomorphic substitution process.

Fig. 2a shows the process of the gradual replacement of Ca^2+^ in CaAl-LDH by Cd^2+^ to form CdAl-LDH. The Gibbs free energy change (Δ*G*) of each step is shown in Fig. 2b. The calculations show that for Ca-6-LDH, Ca-7_OH_-LDH and Ca-7_H_2_O_-LDH, the free energy initially decreases and then increases with each step, and the energy of the final mineralized structure is higher than that of the initial structure. However, in the Ca-5-LDH model, the free energy of each structure during the mineralization process is much lower than that of the initial structure, which indicates that the entire mineralization process in the pentacoordinate structure is energetically favorable. In order to explore the universality of this performance, the mineralization process of Ni^2+^ ions has also been calculated and shown in Fig. S4.† The performance differences during the mineralization of Ni^2+^ ions are consistent with the mineralization of Cd^2+^ ions. The change of Gibbs free energy during isomorphic substitution suggests that the Ca-5-LDH structure with hydroxyl defects is favorable for the mineralization process, and the reasons for this difference will be discussed below.

### Reasons for performance differences

In order to further explore the reasons for the influence of different coordination structures in the LDH structure on the mineralization performance, the weak interaction between Ca^2+^ and the surrounding environment was estimated using the Independent Gradient Model (IGM)^39^ approach. The IGM method supports artificially dividing the model into two fragments, and using the electron density gradient value between the fragments to obtain the δg function, so as to analyze the weak interaction region and its characteristics.^40,41^ Fig. S5a–d† show the color-filled isosurface of the Ca^2+^ and its surrounding coordination groups. It can be seen intuitively that the weak interaction between the Ca^2+^ and the surrounding groups in the pentacoordinated Ca-5-LDH model is the smallest, while the seven-coordinated Ca-7_OH_-LDH and Ca-7_H_2_O_-LDH models have additional weak interactions between the Ca^2+^ and the surrounding seventh groups. The quantitative weak interaction analysis is given in Table S2† in order to further precisely identify the weak interaction. The δg function value is summarized in Fig. S6,† the stronger the interaction between atoms, the larger the δg of the interaction region will be. The δg function values of the Ca-7_OH_-LDH and Ca-7_H_2_O_-LDH models were found to be bigger than those of the Ca-6-LDH and Ca-5-LDH models, and the intensity order represented by δg is consistent with the electron density and potential energy density (Table S2†). However, static weak interaction analysis lacks detailed information on the mineralization process, and provides little understanding of the excellent mineralization performance of Ca-5-LDH. Therefore, AIMD simulation of the Ca-5-LDH structure was conducted to further explore its coordination changes during the mineralization process.

**Fig. 3:**
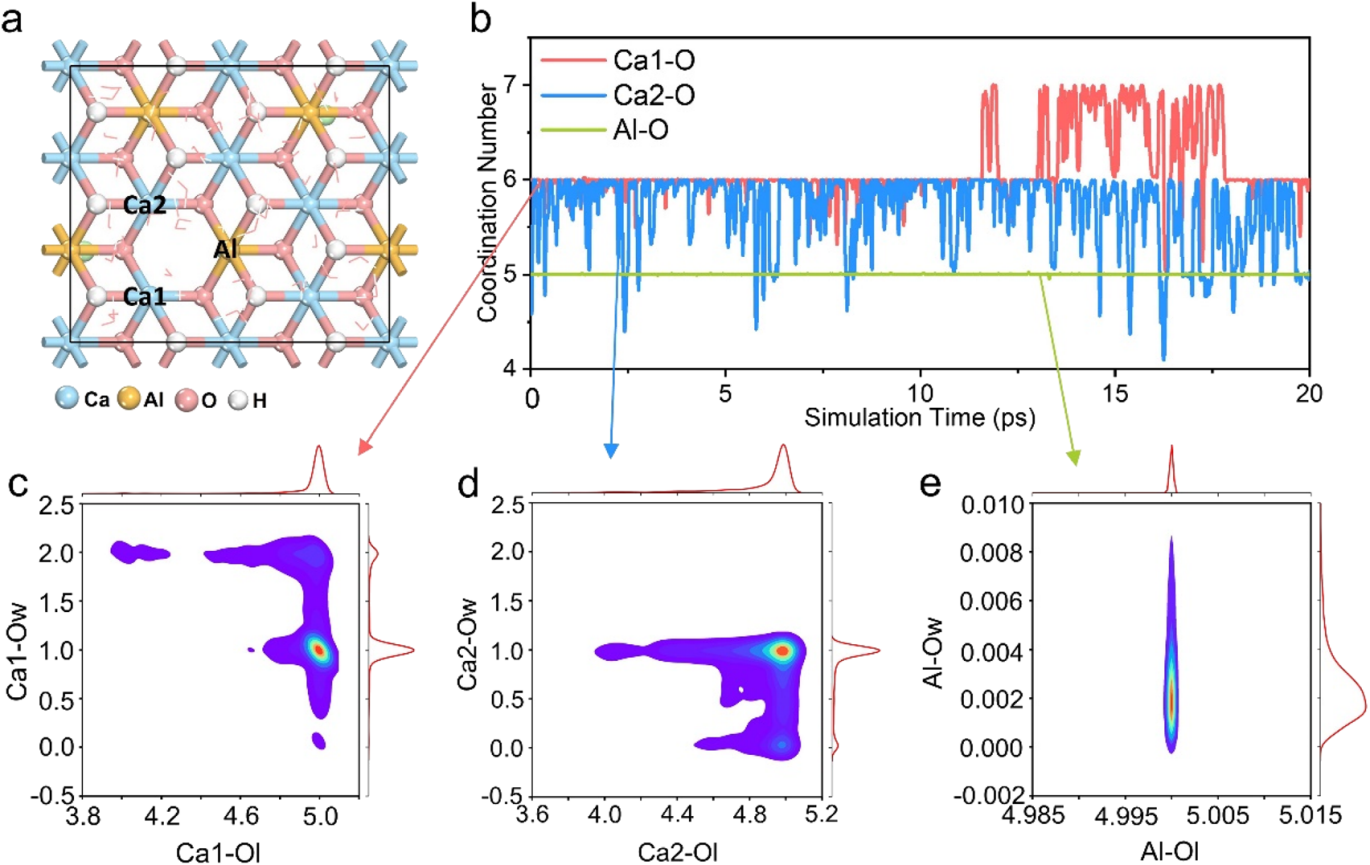
(a) The Ca-5-LDH structure of the 20 ps AIMD simulation, the metal ions around the O vacancy are defined as Ca_1_, Ca_2_, and Al, respectively; (b) evolution of coordination number values of Ca_1_–O, Ca_2_–O and Al–O during the AIMD simulation of the Ca-5-LDH structure; (c) coordination distribution around the Ca_1_ ion, the redder the color, the higher the probability; (d) coordination distribution around the Ca_2_ ion; (e) coordination distribution around the Al ion.

In order to simulate the Ca^2+^/Cd^2+^ cation exchange process in Ca-5-LDH more accurately, we performed a 20 ps AIMD simulation. Fig. 3a shows the structure of the dynamic simulation, and the energy and temperature changes during the simulation process are shown in Fig. S7.† From Fig. 3a, it can be seen that two Ca^2+^ ions (Ca_1_ and Ca_2_) and the Al^3+^ ion (Al) all exhibit a pentacoordinated structure due to the absence of hydroxyl groups. Herein, we quantitatively summarized the changes in coordination numbers of Ca_1_, Ca_2_, and Al with all surrounding oxygen atoms (Ca_1_–O, Ca_2_–O and Al–O) during the simulation process and the results are shown in Fig. 3b. It can be seen that Ca_1_–O and Ca_2_–O exhibit a hexacoordination structure during the AIMD process, which is different from the constructed initial model (pentacoordination). However, it should be noted that the Al^3+^ ion around the defect site always retains pentacoordination with the surrounding O atoms, consistent with the initial structure, which indicates that the laminate has not been totally restored to a hexacoordinated structure during the mineralization process. There are other reasons for the formation of the hexacoordinated structure of Ca^2+^ ions as discussed below.

To further explore the hexacoordinated structure composition of Ca_1_ and Ca_2_ ions during the mineralization process, the O atoms in the system were divided into two types as shown in Fig. S8,† which are LDH layer O atoms (Ol) and intercalated solvent O atoms (Ow). The distribution of these two kinds of O surrounding the Ca_1_ ion is shown in Fig. 3c. It can be seen that the Ca_1_ ion has five-coordination with Ol and one coordination with Ow. The results indicate that during the process of mineralizing, the Ca_1_ ion forms an additional coordination with the O atom in the solution while maintaining a pentacoordination with the hydroxyl groups in the LDH layers. The analysis of the coordination structure around the Ca_2_ ion is shown in Fig. 3d. It can be seen that similar to the coordination structure of the Ca_1_ ion, the Ca_2_ ion also forms coordination structures with both Ol and Ow. That is to say, while maintaining a five-coordinated structure with O in the layers, Ca^2+^ forms additional coordination with O in H_2_O.

The analysis of the coordination structure around Al ions is shown in Fig. 3e; Al forms five-coordination with Ol and basically no coordination with Ow. The coordination distribution around the Ca_1_, Ca_2_, and Al ions suggests that Ca-5-LDH can not only maintain the hydroxyl defects, but also that the Ca^2+^ ions may form additional coordination with O atoms in the solvent environment (Ca-5-LDH-H_2_O). In order to observe these structures, snapshots taken during the AIMD process at 0 ps, 10 ps and 20 ps are shown in Fig. S9.† The Ca-Ol coordination surrounding the Ca^2+^ ions can be clearly seen. Following on from these results, it can be concluded that Ca-5-LDH not only exists stably in the solution environment during mineralization, but also forms additional coordination with H_2_O in solution (Ca-5-LDH-H_2_O).

To investigate the presence of the aforementioned additional coordination during the mineralization process, we examined the initial three structures. These structures correspond to the stages following the introduction of one Cd^2+^ ion (CaAlCd1-5-LDH), two Cd^2+^ ions (CaAlCd2-5-LDH), and three Cd^2+^ ions (CaAlCd3-5-LDH) into the LDH layers (Fig. S10a–S12a†). We conducted AIMD simulations lasting 10 ps, and the results of the coordination number and metal cation distribution analysis during the simulation are presented in Fig. S10–S12.† The coordination behavior for Ca^2+^, Cd^2+^, and Al^3+^ in CaAlCd*x*-5 (*x* = 1, 2 and 3) is found to be similar to that in Ca-5-LDH. That is, Cd^2+^ and Ca^2+^ could form additional bonds with H_2_O during the mineralization process.

Based on the previous analysis of IGM weak interactions and additional coordination phenomena during mineralization, it is reasonable to conjecture that the dissolution of Ca^2+^ ions is optimal for Ca-5-LDH compared with Ca-6-LDH, Ca-7_OH_-LDH and Ca-7_H_2_O_-LDH, while Ca-5-LDH-H_2_O further promotes Ca^2+^ dissolution. Based on this, we calculated the dissolution energy of Ca^2+^ in different models using DFT (Fig. S13†). The energy required for Ca^2+^ dissolution from Ca-7_OH_-LDH and Ca-7_H_2_O_-LDH is higher than that for Ca-6-LDH and Ca-5-LDH. Moreover, it can be seen that Ca-5-LDH-H_2_O facilitates the dissolution of Ca^2+^ compared with Ca-5-LDH. Additional interactions hinder the dissolution of Ca^2+^ in Ca-6-LDH and Ca-7*x*-LDH. Ca-5-LDH forms additional coordination with H_2_O (Ca-5-LDH-H_2_O) and thereby promotes the process of isomorphic substitution of Cd^2+^, which explains the prominent mineralization performance of Ca-5-LDH shown in Fig. 3.

### Experimental verification

In the previous theoretical calculation section, we have proposed a reliable strategy to improve the mineralization performance of CaAl-LDH, that is, by changing the coordination number of the Ca^2+^ ions and constructing an unsaturated coordination LDH structure. To challenge this hypothesis, we set out to prepare low-coordination CaAl-LDH materials containing a large number of hydroxyl defects within the metal hydroxide layers.

**Fig. 4:**
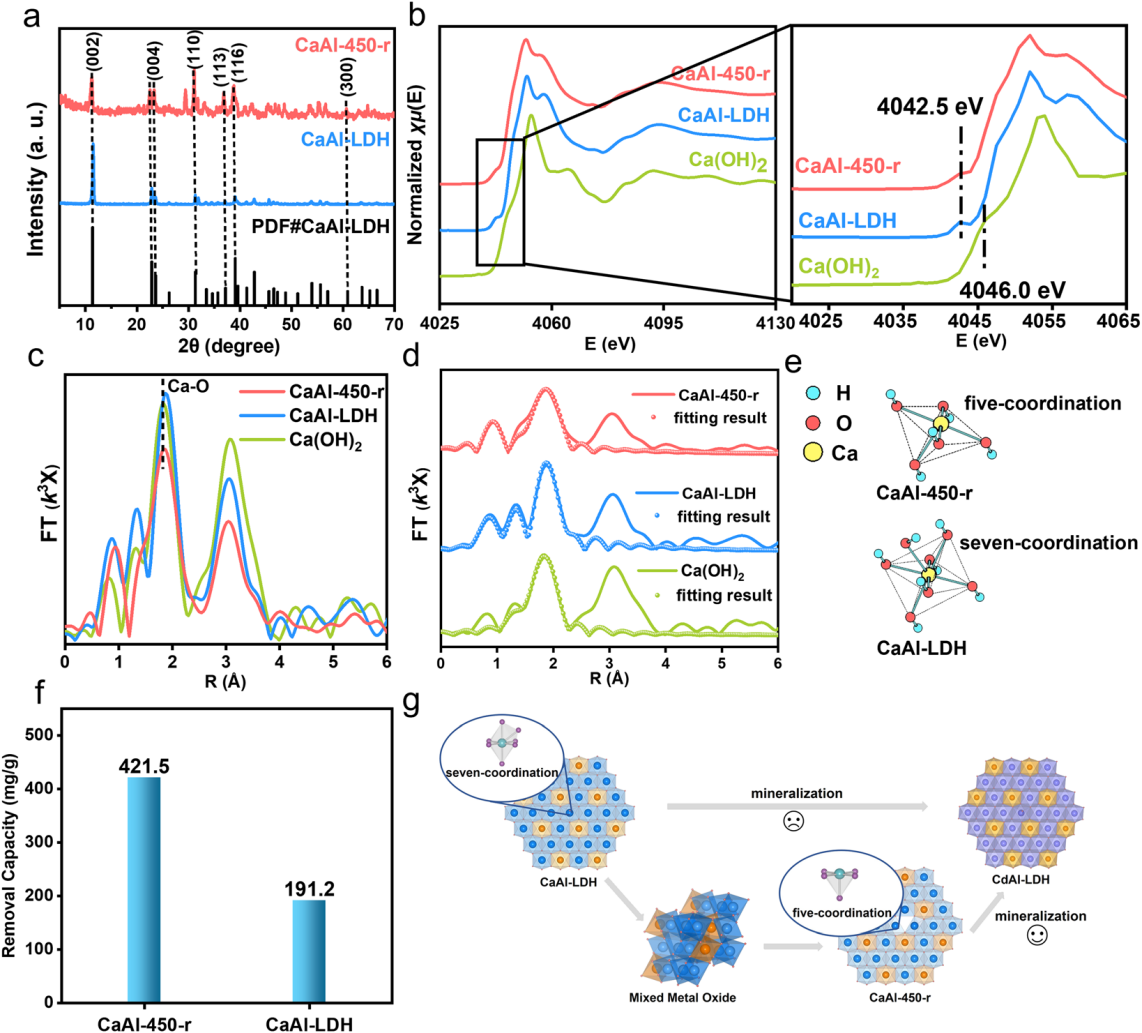
(a) XRD patterns of CaAl-LDH, CaAl-450-r; (b) K-edge XANES spectra of Ca for Ca(OH)_2_, CaAl-LDH, CaAl-450-r; (c) magnitude of *k*^3^-weighted FT spectra of Ca(OH)_2_, CaAl-LDH, CaAl-450-r; (d) fitting results of magnitude of *k*^3^-weighted FT spectra of Ca(OH)_2_, CaAl-LDH, CaAl-450-r; (e) coordination model of Ca^2+^ in CaAl-450-r and CaAl-LDH samples. (f) Mineralization capacities of CaAl-LDH and CaAl-450-r treating 1000 mg L^−1^ Cd^2+^ solution. (g) Schematic illustration of comparison between mineralization processes using CaAl-LDH and CaAl-450-r.

The memory effect of LDHs refers to the regeneration of the layered structure of LDH materials by placing their calcined products (mixed metal oxide, MMO) in an aqueous solution after calcination. This has been previously shown to be a common method for introducing defects into the metal hydroxide layers.^42–44^ We first prepared the CaAl-LDH, and then calcined it at different temperatures (*t*) from 200 °C to 500 °C; the XRD patterns of the synthesized CaAl-LDH and CaAl-*t* (calcination product) are shown in Fig. S14.† The XRD of the pristine CaAl-LDH is consistent with previous reports. The XRD pattern of the calcination products CaAl-*t* shows that at higher temperatures, the LDH layers collapse and transform into mixed oxide phases, including Al_2_O_3_ and amorphous CaO, as observed by the new Bragg reflections in the XRD.^45^ The XRD data (Fig. S14†) indicate that the LDH structure begins to collapse above 300 °C, and phase separation occurs at higher temperatures. CaAl-450 was selected as a typical sample to represent the structure after calcination. The SEM images of the initial CaAl-LDH structure and the CaAl-450 structure are shown in Fig. S15a and b.† The initial CaAl-LDH structure shows a layered morphology with a platelet size of a few micrometers, while the CaAl-450 structure shows an agglomerate caused by the calcination. In order to obtain the defective LDH structure, we reconstructed CaAl-450 in water (CaAl-450-r). As shown in Fig. 4a, the XRD shows the typical Bragg reflections (002), (004) and (110) expected for an LDH, indicating that CaAl-450-r has been fully restored to a crystalline LDH. Moreover, the SEM image of CaAl-450-r (Fig. S15c†) reveals a lamellar morphology.

We used Ca(OH)_2_ as a reference material during the XAFS investigation of the coordination environment in both CaAl-LDH and CaAl-450-r. The results are shown in Fig. 4b, c and S16.† From *E* space analysis (Fig. 4b), comparing Ca(OH)_2_ which contains normally hexacoordinated Ca^2+^, a weaker front-edge peak around 4042.5 eV was observed in both CaAl-LDH and CaAl-450-r, which indicates the presence of a geometrically distorted octahedral Ca(OH)_6_ environment.^46–48^ This suggests a change in the coordination number of Ca^2+^ in both CaAl-LDH and CaAl-450-r. More detailed information regarding the coordination number can be obtained by further analysis of the *K* and *R* space data. Analysis of the *K* space (Fig. S16†) shows that the CaAl-450-r forms an LDH structure after reconstruction. Analysis of the *R* space data (Fig. 4c) shows that there are two main peaks in *R* space for all samples located at ∼1.83 Å and ∼3.08 Å respectively, corresponding to the first shell Ca–O and the Ca–Ca/Al second shell. Furthermore, the first shell peak intensities for CaAl-LDH are strongest in Fig. 4c, followed by the Ca(OH)_2_ sample, and finally the CaAl-450-r sample. To further quantify the analysis of Ca–O coordination numbers, *R*-space data (Fig. 4c) in XAFS characterization results were fitted as shown in Fig. 4d and Table S3.† It should be noted that the coordination number of the first Ca–O shell for CaAl-LDH samples is ∼7.2, much larger than that of the Ca(OH)_2_ reference (∼6.0).^28^ The existence of additional Ca-OH coordination in a solid base catalyst, CaAl-LDH has been previously reported,^28^ while for the CaAl-450-r, the coordination number of the first Ca–O shell is determined to be ∼4.9, lower than that for the Ca(OH)_2_ reference (∼6.0). These data further substantiate the existence of an oxygen defect-containing CaAl-400-r, which results in a distorted Ca(OH)_6_ octahedron in *E* space (Fig. 4b). We have also used electron paramagnetic resonance (EPR) measurements to confirm the existence of the oxygen defects in CaAl-450-r. As shown in Fig. S17,† CaAl-450-r exhibits a strong EPR signal at *g* = 2.003, indicating the formation of oxygen vacancies in CaAl-450-r.^49,50^ A schematic representation of the Ca coordination for CaAl-LDH and CaAl-450-r is shown in Fig. 4e. CaAl-LDH contains an additional coordination structure, while CaAl-450-r presents a coordinatively unsaturated structure with oxygen defects. We compared these Ca-LDHs with different coordination environments for super-stable mineralization of Cd^2+^ ions as discussed below.

The mineralization performance for Cd^2+^ exhibited by CaAl-LDH and CaAl-400-r is shown in Fig. 4f. The methods used to calculate adsorption capacity have been extensively reported.^19,38,44,51^ The mineralization performance of CaAl-450-r, which corresponds to a Ca-5-LDH structure, was 421.5 mg g^−1^, much higher than that of CaAl-LDH (191.2 mg g^−1^), corresponding to the Ca-7_OH_-LDH/Ca-7_H_2_O_-LDH structure, which is consistent with the theoretical calculations. The XRD characterization of the final mineralized product (named CaAl-LDH-Cd and CaAl-450-r-Cd) is shown in Fig. S18.† From the analysis of the XRD characterization, it can be seen that the mineralized product shows an LDH structure, which suggested that the Cd^2+^ replaces Ca^2+^ in the LDH layers, proving that the mineralization mechanism proceeds mainly *via* cation substitution, similar to our previous report (Fig. 4g).^19,38,51^ The SEM images of CaAl-LDH-Cd and the CaAl-450-r-Cd sample are shown in Fig. S19.† The apparent lamellar structure suggested that the sample maintains the LDH structure after mineralizing the Cd^2+^ ions, which is consistent with the XRD data, demonstrating an isomorphic substitution mechanism during the mineralization process. Some previous reports showed that the mechanism of mineralization of heavy metal ions by the LDH material includes isomorphic substitution and surface adsorption, regardless of which, the dissolution of Ca^2+^ can promote the mineralization process.^17^ In order to study the effects of calcination and reconstruction on specific surface areas,^44^ Brunauer–Emmett–Teller (BET) N_2_ adsorption/desorption experiments were performed. As shown in Fig. S20,† CaAl-LDH exhibited a specific BET surface area of 19.23 m^2^ g^−1^, while CaAl-450-r displayed a similar result (24.33 m^2^ g^−1^). These similar surface areas indicate that the improvement in the mineralization performance of Cd^2+^ in the different samples mainly comes from the difference in the coordination structure of Ca^2+^, not a surface area effect.

**Scheme 2:**
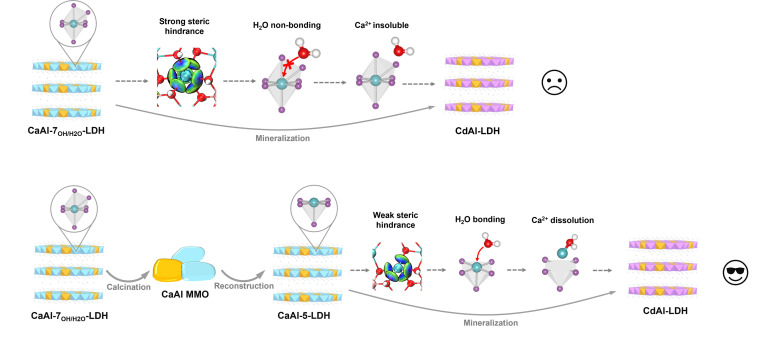
The display of the LDH micro-coordination structure and the explanation of the mechanism for improving the mineralization performance, based on the combination of calculation and experimental results.

Based on the above theoretical and experimental results, the display of the CaAl-LDH micro-coordination structure and the explanation of the mechanism for improved mineralization performance can be summarized in Scheme 2. In the pristine Ca-7_OH_-LDH/Ca-7_H_2_O_-LDH, Ca ions form a seven-coordinate structure surrounded by significant steric hindrance effects that prevent further H_2_O from coordinating with Ca ions and impede Ca dissolution. The pristine Ca-7_OH_-LDH/Ca-7_H_2_O_-LDH was calcined and transformed into CaAl-MMO, and penta-coordinated Ca-5-LDH was obtained after reconstruction through utilizing the memory effect. Compared with Ca-7_OH_-LDH/Ca-7_H_2_O_-LDH, the steric hindrance effect of Ca-5-LDH was weaker, and subsequent additional H_2_O coordination promoted the dissolution of Ca ions and thus promoted the mineralization process of Cd ions.

## Conclusion

In this work, the cation exchange of Ca^2+^ by Cd^2+^ in CaAl-LDH has been investigated using density functional theory (DFT) and *ab initio* molecular dynamics (AIMD) simulations. A significant enhancement in Cd^2+^ mineralization performance within the pentacoordinated CaAl-5-LDH structure was predicted. The unsaturated coordination CaAl-LDH was successfully prepared using a calcination-reconstruction process, and its superior mineralization ability for Cd^2+^ is consistent with our theoretical calculations. This work is of great significance for understanding the mechanism of Ca-LDHs to remove heavy metals and providing guidance for the design of mineralizers with enhanced performance.

## Data availability

The data supporting the findings of this study are available within the article and its ESI.†

## Author contributions

Zixian Li: conceptualization, investigation, writing – original draft. Nuo Xu: experiments, investigation. Jing Ren: XAFs investigation. Haigang Hao: experiments and corresponding analysis. Rui Gao: dynamic simulation. Xianggui Kong: investigation. Hong Yan: investigation. Xiao Hua: investigation. Yung-Kang Peng: investigation, supervision. Shulan Ma: investigation, supervision. Dermot O'Hare: investigation, supervision. Yufei Zhao: conceptualization, supervision, project administration, resources, writing – review & editing.

## Conflicts of interest

The authors declare no conflicts of interest.

## Supplementary Material

SC-015-D4SC02860K-s001
