# Supplementary material for: Theory-driven design of cadmium mineralizing layered double hydroxides for environmental remediation
Source: Chem Sci. 2024 Jul 19;15(32):13021–31. doi: 10.1039/d4sc02860k (PMC11323326; doi:10.1039/d4sc02860k)
Supplement: SC-015-D4SC02860K-s001 [file SC-015-D4SC02860K-s001.pdf]

Electronic Supplementary Material (ESI) for Chemical Science.
This journal is © The Royal Society of Chemistry 2024

Supporting Information

Theory-driven design of cadmium mineralizing layered double hydroxides for environmental remediation

Zixian Li,[a] Nuo Xu,[a] Jing Ren,[a] Haigang Hao,[c] Rui Gao,[c] Xianggui Kong,[a] Hong Yan,[a] Xiao Hua,[d] Yung-Kang Peng,[e] Shulan Ma,[f] Dermot O'Hare,[g] Yufei Zhao*[ab]

[a]State Key Laboratory of Chemical Resource Engineering, Beijing University of Chemical Technology, Beijing 100029, P. R. China. Email: zhaoyufei@mail.buct.edu.cn (Y. Zhao)

[b]Quzhou Institute for Innovation in Resource Chemical Engineering, Quzhou 324000, Zhejiang, P. R. China

[c]College of Chemistry and Chemical Engineering, Inner Mongolia University, 010021 Hohhot, Inner Mongolia, P. R. China

[d]Department of Chemistry, Lancaster University, Lancaster LA1 4YB, United Kingdom

[e]Department of Chemistry, City University of Hong Kong, Hong Kong, Hong Kong SAR 999077, P. R. China

[f]Beijing Key Laboratory of Energy Conversion and Storage Materials and College of Chemistry, Beijing Normal University, Beijing 100875, P. R. China

[g]Chemistry Research Laboratory, Department of Chemistry, University of Oxford, Mansfield Road, Oxford OX1 3TA, United Kingdom

Computational Details

DFT calculation:

The density functional theory calculations were performed with Vienna ab initio simulation package (VASP) code.[1-3] The electron-ion interaction was described by the projector-argumented plane wave (PAW)[4, 5] approach associated with plane-wave basis sets. The Perdew-Burke-Ernzerhof (PBE)[6] functional was used in form of the generalized gradient approximation (GGA). An energy cut-off of 400 eV was employed. In the calculations, the interfacial atoms were relaxed the total energy convergence below 1×10^{-5} eV, and the atomic forces were converged to 0.01 eV/Å in all structural optimizations. A 3×3×1 Monkhorst-Pack k-point mesh was adopted to sample the Brillouin zone.[7, 8]

The model of bulk Ca_2Al-Cl-LDH was built according to the experimental XRD measurement. Therefore, the model bulk Ca_2Al-LDH was constructed with the space group of $P\bar{3}m_1$[9], with the lattice parameters of $a = b = 3.37$ Å, $c = 7.56$ Å, $a = \beta = 90^o$, $\gamma = 120^o$. The supercell of Ca_2Al-Cl-LDH was 9 × 2 × 1 in the *a*-, *b*-, and *c*- directions. Six chlorine anions were put in the interlayer gallery of Ca_2Al-LDH to balance the charge. The exposed facets of LDH were normally the (003) and (110) facets. In the (003) facet, the Ca^{2+} cation was blocked by the hydroxyl group, thus the isomorphic substitution of Ca^{2+} by Cd^{2+} and Ni^{2+} was inhibited by the steric hindrance. Therefore, it was deduced that the cation exchange reaction occurred in the (110) facet of LDH. Then, the (110) facet of Ca_2Al-Cl-LDH was cleaved with a vacuum layer of 15 Å. The series of models for $(CaCd)_2Al$-LDH/$(CaNi)_2Al$-LDH were built in the similar way by substituting Ca^{2+} cation with Cd^{2+}/Ni^{2+} cation.

The model of $Ca^{2+}(aq)$ was built by putting one Ca^{2+} cation in a solvent box containing 33 water molecules with a density of 1.0 g/cm^2. Two chlorine anions were also put in the solvent box to keep the model neutral.[10] The model of $Cd^{2+}(aq)$/$Ni^{2+}(aq)$ was built in the similar way by replacing Ca^{2+} with Cd^{2+}/Ni^{2+}.

AIMD simulation:

All the AIMD simulations reported in this work were performed within the density functional theory (DFT) framework according to the generalized gradient approximation (GGA) using the Perdew−Burke−Ernzerhof (PBE) functional, which was implemented in the CP2K/Quickstep code.[10] Core electrons were described by Goedecker−Teter−Hutter (GTH) pseudopotentials[11, 12] and valence electrons were described by a mixed Gaussian and plane waves basis (GPW). The wave functions were expanded on a double-ζvalence polarized (DZVP) basis set[13] along with an auxiliary plane-wave basis set at a cutoff energy of 400 Ry. The Brillouin zone was sampled by the gamma approximation. During AIMD, the nuclei were treated within the Born-Oppenheimer approximation with a time step of 1 fs for equilibrium simulation. The temperature was maintained at 298.15 K (25 °C) using a CSVR thermostat coupled to the system with a time constant of 300 fs in the Canonical ensemble (NVT)[14, 15]. The convergence criterion for the self-consistent fields was set to 10^{-6} Hartree.

Ca_2Al-LDH with the metal ratio of 2:1 was constructed; The supercell consists of 2×6×1 crystallographic unit cells. 4 chlorine anions and 50 H_2O molecules were put in the interlayer gallery to simulate solution environment with a density of 1.0 g/cm^2.

Coordination number calculation:

Coordination number (CN) was characterized as collective variables (CVs) to monitor the dissolution process. The CN(Ca–Ol) is the coordination number of the Ca ion with all oxygen ions from the LDH lattice, while CN(Ca–Ow) is the coordination number of the Ca ion with all oxygen from water molecules. The CN have the expression as follows as defined in the PLUMED code[16]:

$$CN(Ca \cdot Ol/w) = \sum_{j \in O_{L/S}} S_{ij}(r_{ij}) = \sum_{j \in O_{L/S}} \frac{1 - (\frac{r_{ij} - d_0}{r_0})^n}{1 - (\frac{r_{ij} + d_0}{r_0})^m} \quad (1)$$

Here, we define d_0 is 2.42 Å[17], which is the equilibrium bond length between the Ca^{2+} and O ions; r_0 is 0.4 Å, which is around half of the full width at half maximum of the radial distribution function of Ca–O and n and m are 6 and 12[18], respectively.

Independent gradient model (IGM) analysis:

IGM analysis[19] was performed on the wave function file generated by cp2k calculation using Multiwfn software[20] to explore the weak interaction in the system. The fundamentals for calculating the IGM with the Multiwfn program for weak interaction analysis is briefly discussed below.[21, 22]

δg can be divided into δg_intra for intra-fragment interactions and δg_inter for inter-fragment interactions:

$$g^{inter}(r) = \left| \sum_{A} \sum_{i \in A} \nabla \rho_i(\mathbf{r}) \right| \quad (2)$$

$$g^{IGM,inter}(r) = \left| \sum_{A} \text{abs} \sum_{i \in A} \nabla \rho_i(\mathbf{r}) \right| \quad (3)$$

$$\delta g^{inter}(\mathbf{r}) = g^{IGM,inter}(\mathbf{r}) - g^{inter}(\mathbf{r}) \quad (4)$$

$$\delta g^{intra}(\mathbf{r}) = \delta g(\mathbf{r}) - \delta g^{inter}(\mathbf{r}) \quad (5)$$

i is the atomic number, $\nabla\rho$ is the gradient vector, abs($\nabla\rho$) means to take the absolute value of each component of the $\nabla\rho$ vector inside, | | means to take the modulus of the vector. A is the number of the defined fragment.

Experimental Methods:

Synthesis of CaAl-LDH:

A 40.0 mL solution composed of 13 mmol $CaCl_2$ and 6.5 mmol $AlCl_3 \cdot 6H_2O$ was added drop by drop to a 10.0 mL solution. Simultaneously, NaOH solution (1 mol/L)

was added dropwise to maintain the system at a pH value of 12.3 under magnetic stirring at room temperature. The precipitations, denoted as CaAl-LDH, were collected by washing, centrifugation, and drying.

Synthesis of CaAl-*x*:

The calcined samples CaAl-*x* (*x* refers to the different calcination temperatures: 200, 300, 400, 450 and 500 °C) were obtained under an ambient atmosphere ranging from 200 to 500°C for 3 h (2°C min^{-1}), respectively.

Heavy metal adsorption studies:

The removal experiments were carried in 40 mL Cd^{2+} ions solutions with 1 g/L CaAl-LDH in round-bottom flask under magnetic stirring (400 rpm/min) at room temperature. The concentrations of Cd^{2+} ions were analyzed by inductively-coupled plasma optical-emission spectrometry (ICP-OES) and inductively-coupled plasma-mass spectrometry (ICP-MS). The removal efficiency of Cd^{2+} ions (R %) was calculated by the following equation: $R = (C_0 - C_e) / C_0 \times 100\%$, where the C_0 and C_e was the initial and the equilibrium concentration of Cd^{2+} ions, respectively. The removal capacity of Cd^{2+} ions (q, mg/g) was calculated by the following equation: $q = (C_0 - C_e) \times V / m$, where V (L) and m (g) represent the volume of the suspension and the mass of LDHs, respectively[23, 24].

Characterisation:

X-ray diffraction (XRD) patterns were characterized by a Rigaku XRD-6000 diffractometer equipped with a Cu Kα radiation (λ = 1.5405 Å). Scanning electron microscopy (SEM) images were obtained on the Zeiss Supra 55 SEM equipped with an EDX detector. X-ray absorption near edge structure (XANES) spectrum and extended X-ray absorption fine-structure (EXAFS) spectrum were collected at the 1W1B beamline of Beijing Synchrotron Radiation Facility. The measurements were carried out in a transmission mode at room temperature. EXAFS shell fitting was carried out with Artemis Software.

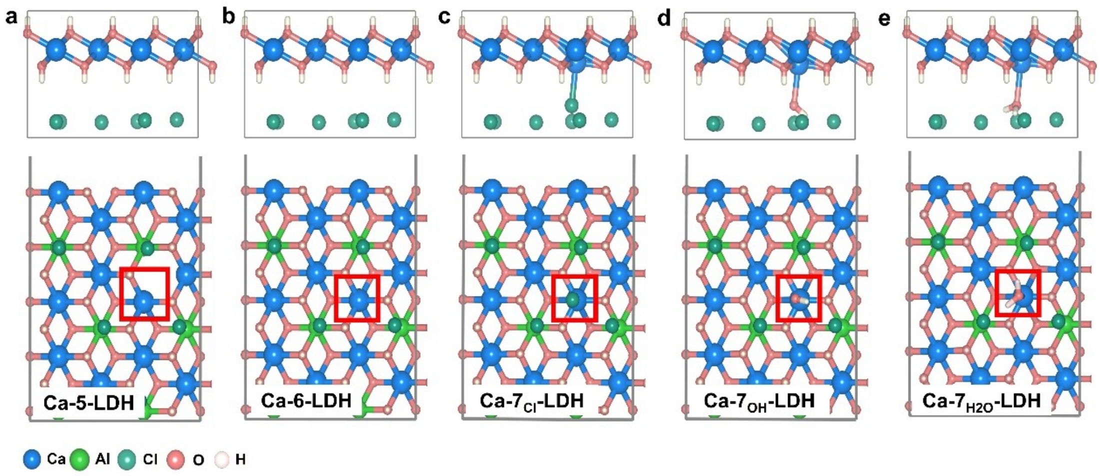

Figure S1. Theoretical models of (a) Ca-5-LDH; (b) Ca-6-LDH; (c) Ca-7_{Cl}-LDH; (d) Ca-7_{OH}-LDH; (e) Ca-7_{H2O}-LDH from top and side view, respectively.

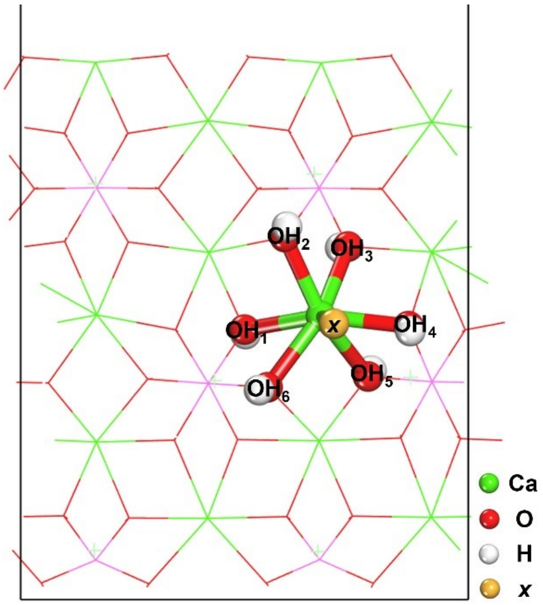

Figure S2. The optimized Ca-7_x-LDH structure shows that the Ca atom is surrounded by six hydroxyl groups, and the x atom represents the seventh coordination group, Cl^-, OH^- and H_2O.

Table S1. Bond length (Å) of Ca^{2+} and the surrounding ligands in Ca-7_x-LDH structures. x represents the seventh ligand.

	Ca-OH_1	Ca-OH_2	Ca-OH_3	Ca-OH_4	Ca-OH_5	Ca-OH_6	Ca-x
x = Cl	2.379	2.494	2.462	2.331	2.352	2.434	3.192
x = OH	2.383	2.536	2.529	2.333	2.350	2.484	2.524
x = H_2O	2.378	2.537	2.496	2.345	2.363	2.480	2.492

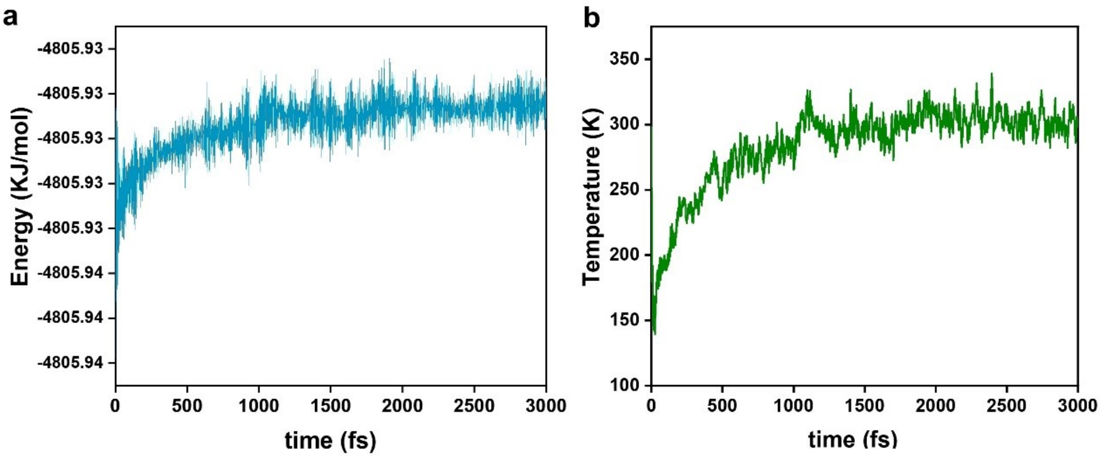

Figure S3. (a) Energy and (b) temperature changes for the 3000 fs AIMD process of Ca-6-LDH structure.

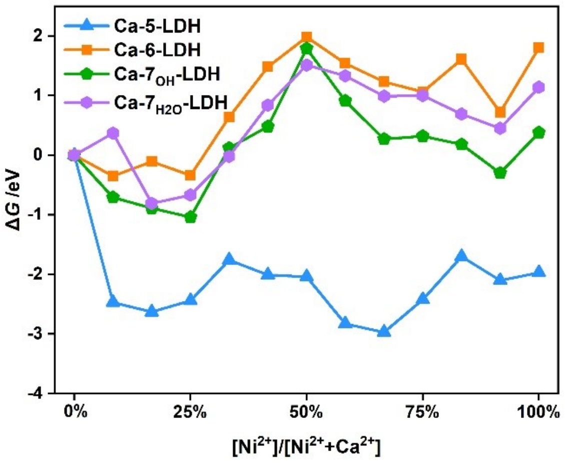

Figure S4. Relationship between the Gibbs free energy and the ratio of [Ni^{2+}] to [Ni^{2+} + Ca^{2+}] in the isomorphic substitution process.

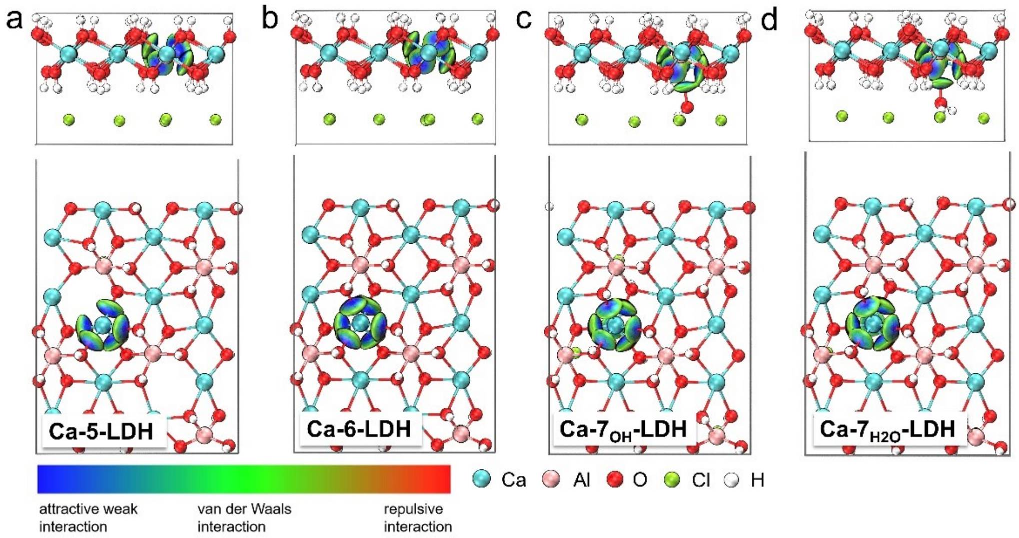

Figure S5. The IGM analysis of weak interactions for (a) Ca-5-LDH; (b) Ca-6-LDH; (c) Ca-7_{OH}-LDH and (d) Ca-7_{H2O}-LDH.

Table S2. Quantitative weak analysis of the Ca^{2+} and surrounding coordination groups

	Ca-5-LDH	Ca-6-LDH	Ca-7_{OH}-LDH	Ca-7_{H2O}-LDH
Density of all electrons	1.889	2.141	2.207	2.224
Potential energy density V(r)	-1.996	-2.167	-2.193	-2.213
δg	4.156	4.74	4.843	4.894

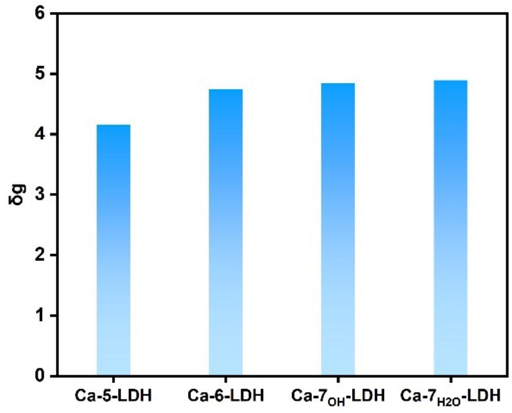

Figure S6. δg of Ca-5-LDH, Ca-6-LDH, Ca-7_{OH}-LDH and Ca-7_{H2O}-LDH.

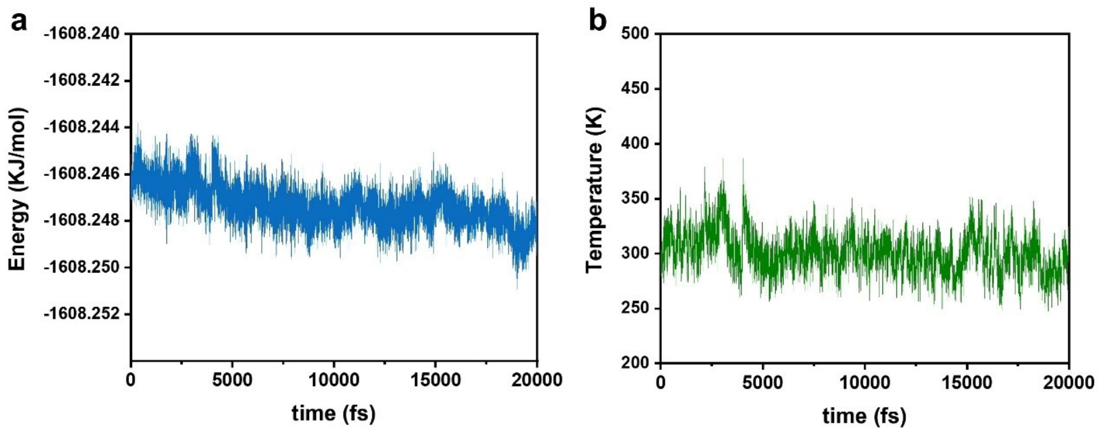

Figure S7. (a) Energy and (b) temperature changes for the AIMD process of Ca-5-LDH structure.

From the changes in system energy and temperature during the simulation process, it can be seen that the dynamic simulation has reached an equilibrium state.

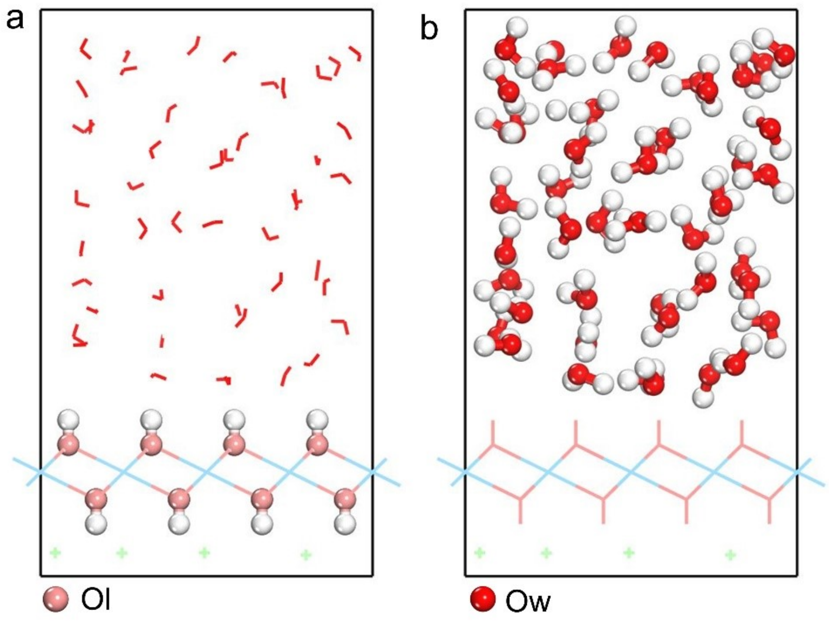

Figure S8. The O atoms in the AIMD system were divided into two types, which are (a) LDH laminate O atoms (named Ol) and (b) solvent environment O atoms (named Ow).

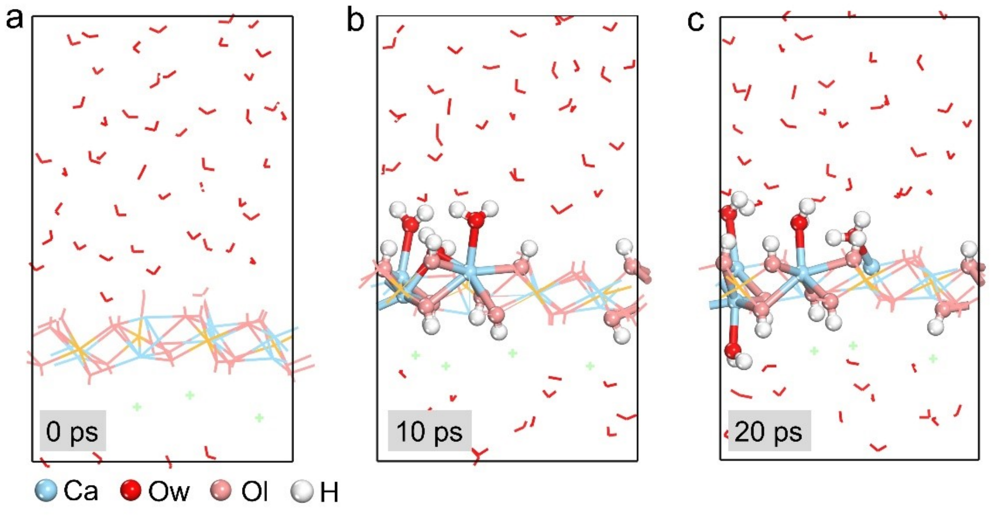

Figure S9. Structures during the AIMD process of Ca-5-LDH at (a) 0 ps, (b)10 ps and (c) 20 ps.

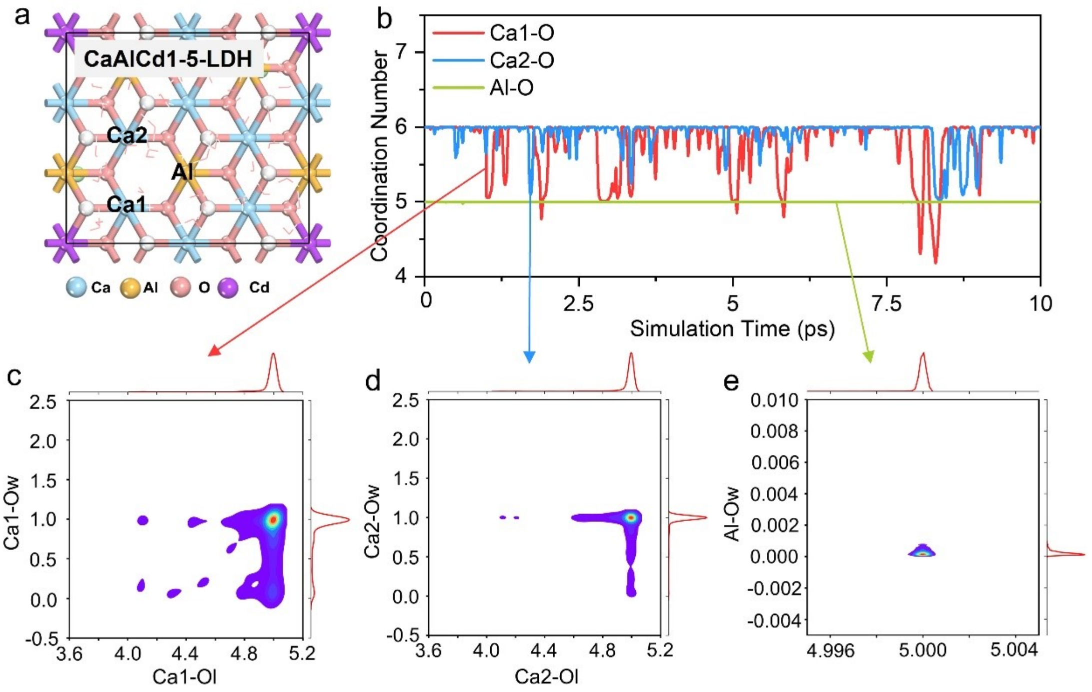

Figure S10. (a) Structures of CaAlCd1-5-LDH in the AIMD simulation; (b) Evolution of coordination number values; (c) Coordination distribution around Ca1 ion, the redder the color, the higher the probability; (d) Coordination distribution around Ca2 ion; (e) Coordination distribution around Al ion.

The CaAlCd1-5-LDH structure was constructed to simulate the initial stage of the mineralization process. Similar to the Ca-5-LDH structure, Figure S10b shows that Ca1-O and Ca2-O exhibit a hexacoordination structure, yet the Al iron around the defect site always keeps pentacoordination with the surrounding O atoms. The distribution of the two kinds of O surrounding Ca1, Ca2 and Al ions is shown in Figure S10c-e. The Ca^{2+} ions have five coordinations with Ol and one coordination with Ow, Al^{3+} forms five coordinations with Ol and basically no coordination with Ow. The results indicate that during the process of mineralizing, Ca^{2+} ions form additional coordination with the O atom in the solution while maintaining pentacoordination with the hydroxyl groups on the laminates.

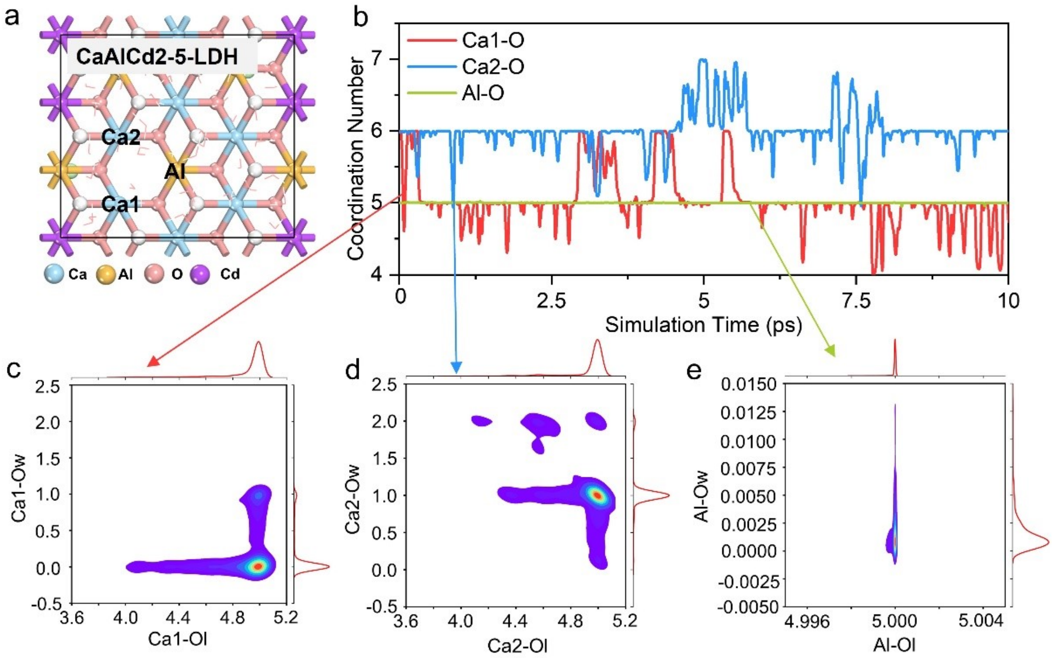

Figure S11. (a) Structures of CaAlCd2-5-LDH in the AIMD simulation; (b) Evolution of coordination number values; (c) Coordination distribution around Ca1 ion, the redder the color, the higher the probability; (d) Coordination distribution around Ca2 ion; (e) Coordination distribution around Al ion.

The CaAlCd2-5-LDH structure was constructed to simulate the initial stage of the mineralization process. Figure S11c-e shows the Ca^{2+} ions have five coordinations with Ol and one coordination with Ow; Al^{3+} forms five coordinations with Ol and basically no coordination with Ow.

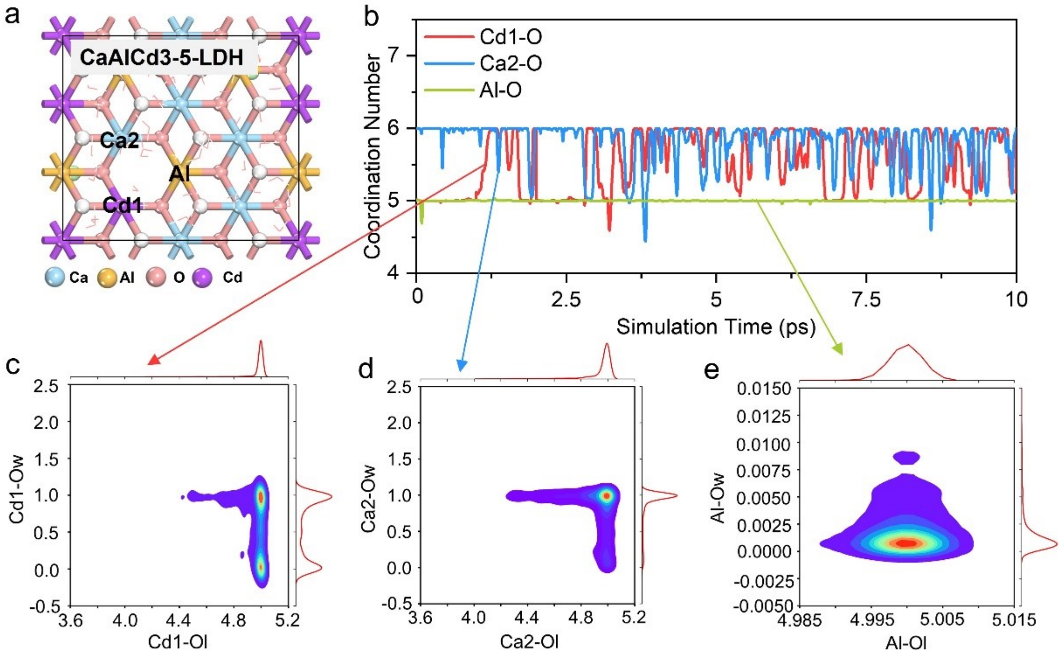

Figure S12. (a) Structures of CaAlCd3-5-LDH in the AIMD simulation; (b) Evolution of coordination number values; (c) Coordination distribution around Cd1 ion, the redder the color, the higher the probability; (d) Coordination distribution around Ca2 ion; (e) Coordination distribution around Al ion.

The CaAlCd3-5-LDH structure was constructed to simulate the initial stage of the mineralization process. Figure S12c-e shows the Ca^{2+} and Cd^{2+} ions have five coordinations with Ol and one coordination with Ow; Al^{3+} forms five coordinations with Ol and basically no coordination with Ow.

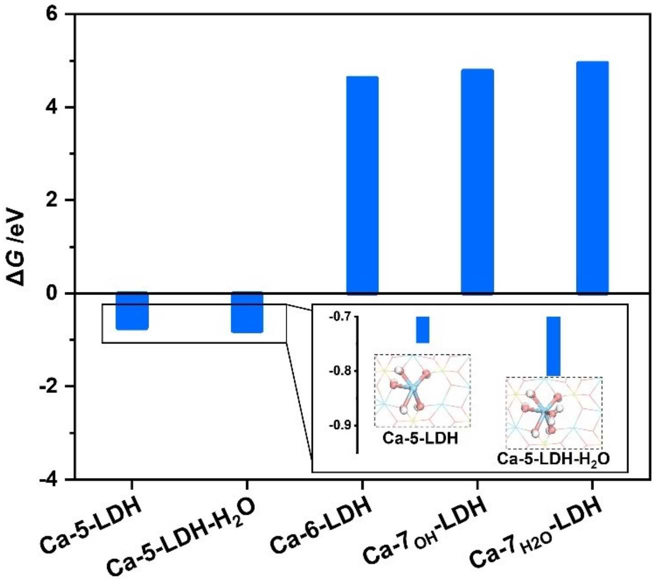

Figure S13. Dissolution energy of Ca^{2+} in different models. The inset shows the structures of Ca-5-LDH and Ca-5-LDH-H_2O.

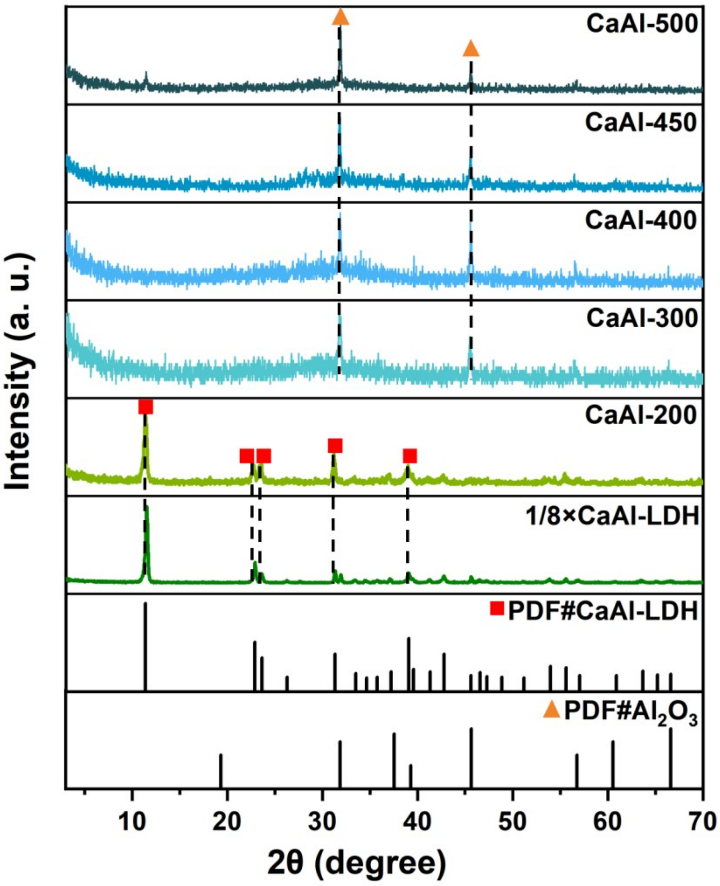

Figure S14. XRD patterns of CaAl-LDH and the obtained MMO structure obtained at calcination temperatures from 200 to 500 °C, respectively.

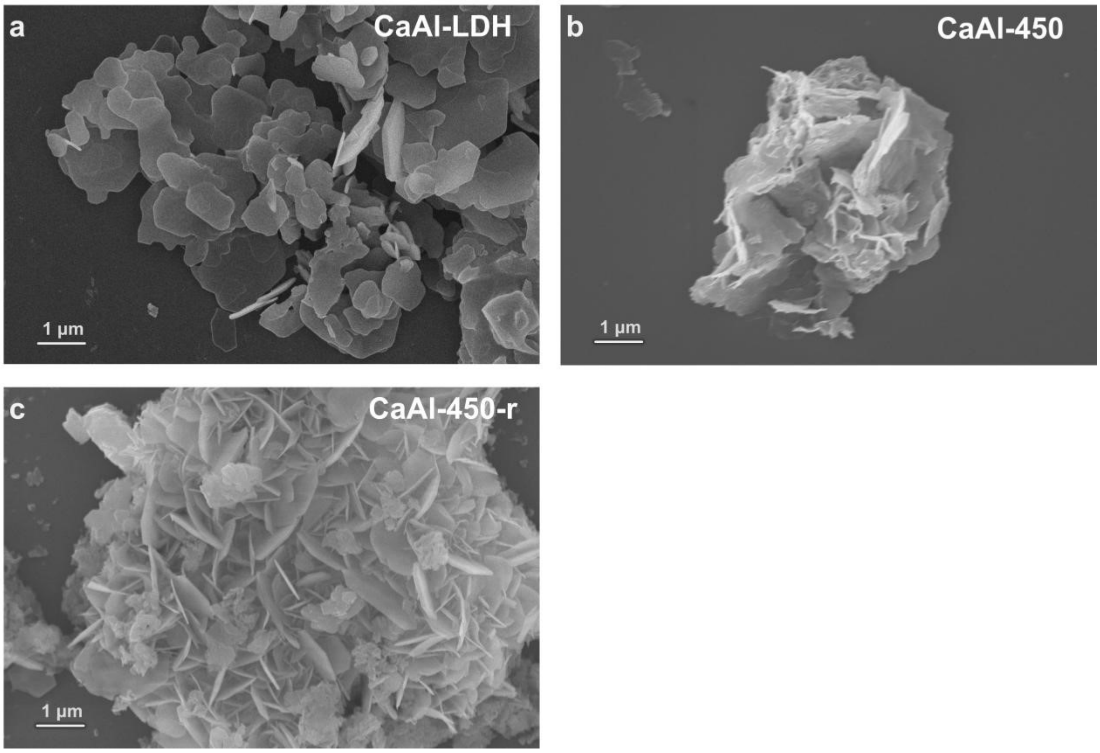

Figure S15. SEM images of (a) CaAl-LDH, (b) CaAl-450 and (c) CaAl-450-r.

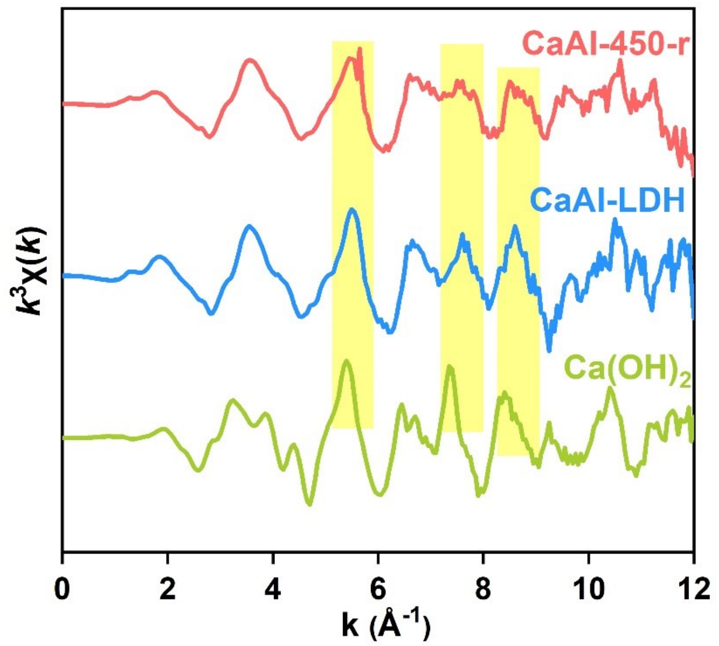

Figure S16. k-space EXAFS oscillation functions k^3 of Ca for $Ca(OH)_2$, CaAl-LDH and CaAl-450-r, respectively.

Table S3. Local structure parameters of Ca-O shell estimated by EXAFS analysis.

Sample	*N*[a]	*R* [Å][b]	σ^2 [10^{-3} Å^2][c]	R-factor [10^{-2}]
$Ca(OH)_2$	6.0	2.34	5.8	0.7
CaAl-LDH	7.2	2.39	9.0	1.6
CaAl-400-r	4.9	2.37	6.0	6.4

[a]N= coordination number; [b]R= distance between absorber and backscatter atoms; [c]σ^2= Debye-Waller factor.

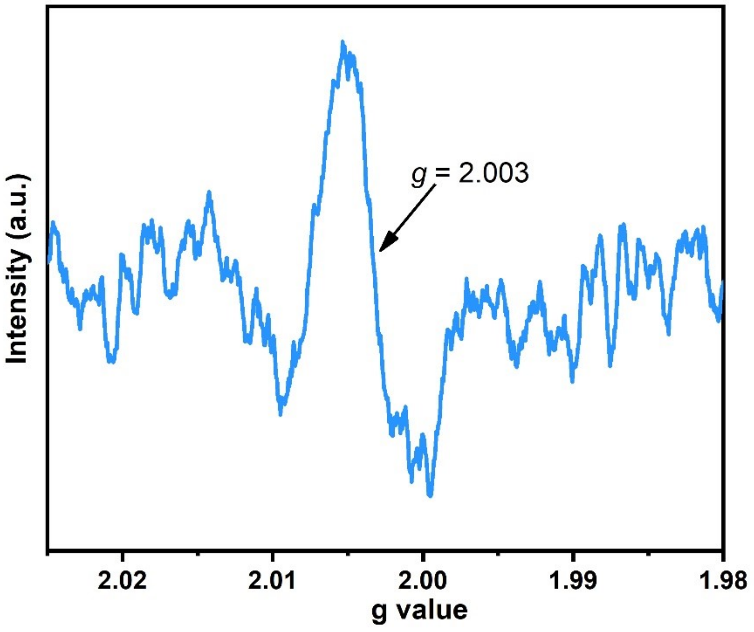

Figure S17. EPR spectra of CaAl-450-r.

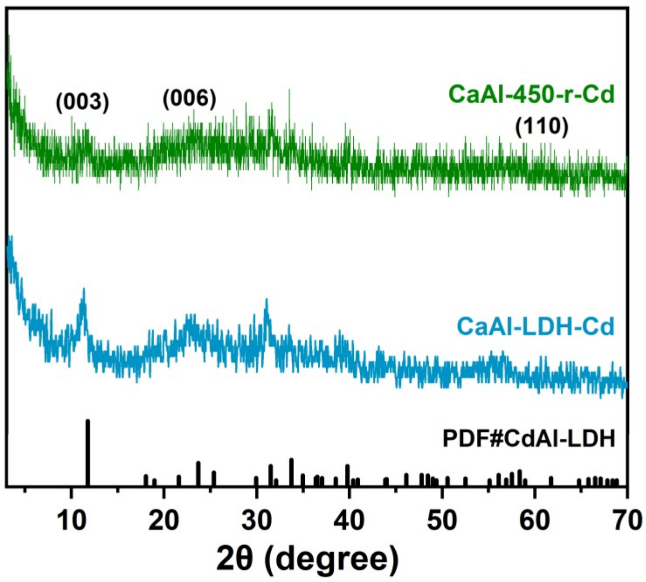

Figure S18. XRD patterns of CaAl-LDH-Cd and the CaAl-450-r-Cd products formed by Cd^{2+} adsorption.

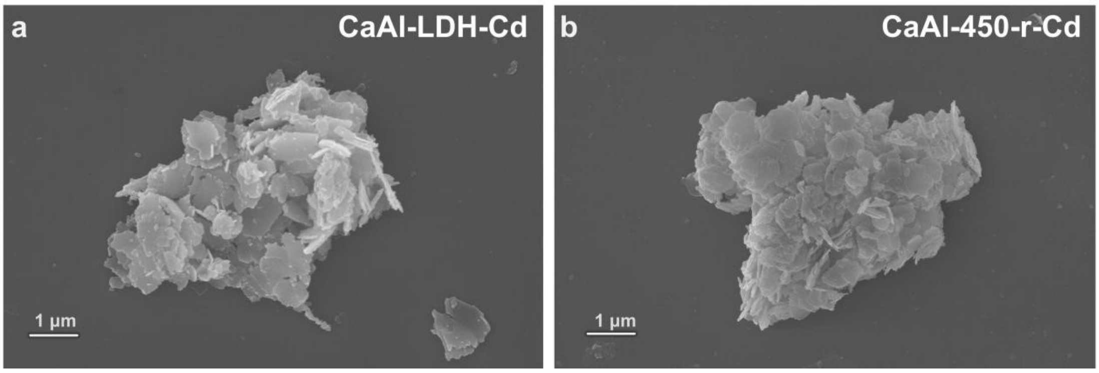

Figure S19. SEM images of (a) CaAl-LDH-Cd and (b) CaAl-450-r-Cd.

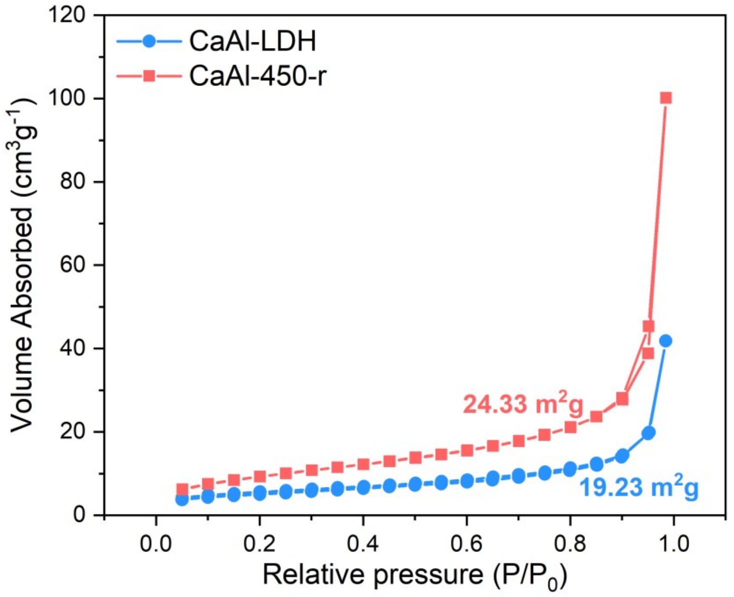

Figure S20. N_2 BET adsorption−desorption isotherms of CaAl-LDH and CaAl-450-r, respectively.

Ca-5-LDH
1.0

7.8200001717	0.0000000000	0.0000000000
0.0000000000	11.7086639404	0.0000000000
0.0000000000	0.0000000000	28.5200004578

H	O	Al	Cl	Ca
34	34	6	6	12

Direct

0.936652005	0.526503980	0.151695997
0.948325992	0.769650996	0.334080994
0.967899978	0.999534011	0.500207007
0.923931003	0.434184998	0.008928000
0.949109972	0.703656018	0.200315997
0.945554972	0.957737982	0.378053010
0.429464996	0.892288029	0.334724993
0.416907996	0.168819994	0.498952001
0.420520008	0.395918995	0.069600001
0.433521003	0.642028987	0.241815001
0.431805998	0.892108023	0.421826988
0.919867992	0.941461027	0.010630000
0.946048021	0.207972005	0.200624004
0.943485975	0.448125005	0.378015995
0.951281011	0.276944011	0.071149997
0.952742994	0.516542017	0.245872006
0.948940992	0.774159014	0.422268987
0.454160988	0.218594998	0.007973000
0.430487990	0.467732996	0.201703995
0.434291989	0.707817972	0.379294008
0.432231992	0.143246993	0.153851002
0.428039998	0.391411006	0.333826989
0.416548014	0.667356014	0.501664996
0.955740988	0.776184022	0.070372000
0.946358025	0.022174999	0.243989006
0.946142972	0.275236011	0.419537008
0.944792986	0.021116000	0.153136000
0.966513991	0.496959001	0.499293000
0.419687986	0.888486028	0.072001003
0.429720998	0.145247996	0.242982998
0.425785989	0.392538995	0.424392015
0.455971986	0.720911980	0.009603000
0.430537999	0.960245013	0.200665995
0.433486015	0.207911998	0.377370000
0.811757028	0.517988026	0.147850007

0.825021982	0.761592984	0.328492999
0.844866991	0.991941988	0.497442991
0.800246000	0.446823001	0.012740000
0.824827015	0.719779015	0.201405004
0.821882010	0.971698999	0.376464993
0.552353978	0.901337981	0.328525990
0.540799022	0.172643006	0.497884005
0.546420991	0.407844007	0.074630000
0.556690991	0.653913021	0.247861996
0.554651022	0.903901994	0.427810013
0.797164977	0.952678025	0.016154001
0.822207987	0.223083004	0.200700000
0.820967972	0.466776997	0.375923991
0.826655984	0.259611994	0.076385997
0.827659011	0.507384002	0.251087010
0.826104999	0.762045979	0.428171009
0.575541019	0.209455997	0.015363000
0.553218007	0.447234988	0.201294005
0.557727993	0.692896008	0.377644002
0.555721998	0.151500002	0.148068994
0.551935971	0.402227998	0.329154015
0.539364994	0.675675988	0.498488992
0.830722988	0.759177983	0.076084003
0.822670996	0.011783000	0.248749003
0.823718011	0.261776000	0.426398993
0.820941985	0.013445000	0.147938997
0.842989981	0.490750015	0.497658998
0.544564009	0.904947996	0.077051997
0.553413987	0.153842002	0.248664007
0.549934030	0.405176997	0.430115014
0.579482019	0.712599993	0.014380000
0.553979993	0.943603992	0.201013997
0.558091998	0.193891004	0.376619011
0.686097980	0.330211014	0.035542998
0.700724006	0.577858984	0.203511000
0.690316975	0.832145989	0.378271997
0.686273992	0.832490027	0.036894001
0.689037979	0.083102003	0.199028999
0.677278996	0.336382002	0.381316006
0.179787993	0.574644029	0.208408996
0.179110005	0.343311012	0.043439001
0.190459996	0.832301974	0.382463008
0.197580993	0.335117996	0.389375001

0.189710006	0.083939999	0.200193003
0.184997007	0.833727002	0.042918000
0.700964987	0.580271006	0.069871001
0.688687980	0.832762003	0.260800987
0.692336023	0.082309000	0.439319015
0.681544006	0.333802998	0.144611999
0.689191997	0.582391977	0.319745988
0.690105975	0.834555984	0.493768007
0.687409997	0.081900001	0.075770997
0.686042011	0.331050009	0.260890990
0.689696014	0.583626986	0.440596998
0.691390991	0.832876027	0.145239994
0.684861004	0.079986997	0.318551004
0.693223000	0.331182986	0.494475007

Ca-6-LDH
1.0

7.8200001717	0.0000000000	0.0000000000
-0.0000015591	11.7086601257	0.0000000000
0.0000000000	-0.0000037977	28.5200004578

H	O	Al	Cl	Ca
36	36	6	6	12

Direct

0.943504930	0.520259380	0.153142899
0.948552728	0.769958794	0.334239990
0.964874804	0.998233914	0.499442786
0.919300616	0.441641301	0.009999700
0.948260427	0.702563524	0.200356394
0.945316672	0.955669224	0.377930313
0.432599813	0.643543184	0.153124705
0.429945886	0.892755628	0.334159195
0.417144686	0.167254895	0.499917597
0.419384807	0.392529488	0.071019299
0.430757701	0.644827783	0.242840797
0.430273503	0.890443206	0.420962214
0.920445919	0.940961480	0.008949000
0.948370278	0.205388099	0.200311705
0.945595384	0.454995990	0.377941906
0.952374101	0.278060794	0.071171798
0.945959210	0.519775808	0.242658496
0.949043572	0.773905098	0.420883596
0.457388401	0.220785305	0.007028100
0.427897811	0.461421192	0.200643197

0.433224410	0.708988070	0.378289312
0.432708204	0.144778103	0.153794095
0.429817408	0.392431110	0.334407598
0.417175710	0.666594803	0.500038087
0.954900622	0.773843288	0.071212299
0.946925879	0.022573501	0.242989704
0.949603677	0.272481412	0.420603991
0.944478393	0.022695299	0.153169304
0.948297501	0.269256800	0.334093392
0.965300679	0.498591304	0.499710113
0.422182500	0.888317406	0.071412101
0.430820495	0.146287307	0.242529601
0.431117088	0.389346987	0.421302110
0.456177890	0.722025990	0.009195900
0.429757893	0.962392092	0.200694606
0.433030397	0.208690003	0.378367096
0.819837272	0.513418078	0.147498697
0.825604379	0.761420786	0.327978492
0.841603518	0.991401911	0.497211307
0.795974791	0.452039301	0.014859200
0.825174689	0.720692873	0.200644299
0.821986794	0.971240282	0.376229703
0.556192577	0.651220500	0.147499993
0.552832127	0.901981592	0.327941000
0.540515304	0.173289299	0.497678787
0.544628680	0.405750602	0.076274298
0.554314494	0.654480815	0.248133302
0.552982628	0.902836323	0.427258104
0.798578799	0.951587498	0.015328200
0.824735522	0.222000495	0.200612202
0.822394609	0.470910490	0.376228690
0.828434825	0.259278595	0.076021701
0.822376728	0.510640800	0.248088807
0.826336682	0.761533201	0.427208811
0.577859282	0.211991906	0.015133500
0.551176190	0.443983793	0.200835705
0.556468427	0.692877710	0.376549810
0.556208074	0.151311606	0.147579506
0.552596211	0.402122796	0.328182608
0.540440321	0.673113585	0.497622401
0.830055773	0.758734703	0.076386303
0.823569179	0.011436800	0.248041198
0.826798797	0.261006594	0.427141309

0.821005523	0.014170700	0.147554293
0.825241625	0.261256009	0.327919900
0.842154920	0.491467893	0.497165799
0.546654880	0.905478001	0.076298498
0.554439723	0.154593095	0.248151496
0.553749681	0.402460307	0.427420110
0.578604281	0.712297082	0.015162300
0.552742600	0.944450319	0.200872004
0.556336701	0.192689896	0.376571894
0.686104000	0.332663894	0.036431801
0.688011408	0.582338214	0.198794305
0.689459503	0.832009673	0.377292901
0.688510418	0.831789792	0.036660701
0.689009488	0.083183400	0.198852107
0.689643025	0.331707299	0.377330810
0.187750503	0.582025170	0.200164497
0.179069102	0.347453386	0.043560900
0.189612001	0.831287503	0.381670505
0.190046206	0.328646898	0.381982088
0.189246103	0.089081600	0.199558794
0.190075502	0.828411877	0.043700401
0.688499510	0.582043707	0.075526901
0.690230310	0.832406580	0.260398895
0.689694583	0.082172699	0.438204199
0.686760128	0.332706392	0.145290494
0.688169420	0.582071185	0.318099409
0.690333724	0.832503498	0.492292315
0.688446581	0.082232498	0.075935103
0.688826501	0.332469612	0.260449111
0.690079391	0.582126021	0.438225001
0.688732505	0.832354426	0.145428196
0.687563181	0.082163103	0.318080395
0.692032218	0.332089812	0.492325097

Ca-7$_{Cl}$-LDH
1.0

7.8200001717	0.0000000000	0.0000000000
-0.0000031182	11.7086601257	0.0000000000
-0.0000037977	-0.0000075953	28.5200004578

H	O	Al	Cl	Ca
36	36	6	7	12

Direct

0.938610077	0.524301827	0.153294995

0.946210504	0.768440306	0.333628714
0.963781416	0.000765700	0.499815494
0.920378089	0.441453815	0.009658300
0.943405092	0.695040107	0.203182295
0.945434809	0.953455806	0.379058987
0.431559712	0.652830124	0.150804102
0.429854095	0.894575000	0.335000813
0.419540614	0.169081897	0.501412988
0.420338809	0.389252692	0.070960499
0.421531111	0.643479824	0.242705896
0.429680288	0.889386177	0.421612203
0.918169677	0.946941197	0.008579500
0.923938394	0.221819699	0.210804403
0.940134287	0.454984814	0.379792213
0.952980220	0.274469197	0.070763998
0.920676112	0.494602799	0.243206903
0.947899401	0.772012830	0.421240389
0.456160486	0.222175196	0.007587300
0.410843492	0.463750690	0.193515599
0.432255685	0.707721293	0.378609896
0.429023504	0.150047898	0.153553307
0.412142485	0.404612601	0.340826392
0.420100212	0.661587179	0.504217386
0.959856689	0.774245679	0.069056503
0.944135785	0.017882200	0.242749304
0.946556926	0.272582293	0.418502808
0.941750526	0.032322299	0.151666805
0.922597229	0.285639703	0.321857601
0.966656804	0.501491308	0.500514388
0.425528795	0.886141598	0.072348803
0.421315700	0.127807394	0.246536896
0.430792391	0.390094787	0.425352186
0.457760394	0.723698020	0.008146600
0.431645989	0.950703025	0.197217196
0.427838594	0.207064003	0.378230602
0.815660715	0.515030324	0.147133306
0.823119819	0.760145128	0.327756286
0.840944290	0.992296576	0.496935189
0.797639072	0.451612085	0.015157100
0.821104884	0.716529429	0.202504307
0.822366774	0.969591916	0.376662999
0.555847883	0.656296194	0.146278694
0.552598000	0.902178526	0.328340292

0.542931199	0.172549605	0.498495013
0.545087576	0.405185997	0.075950503
0.545174181	0.652294397	0.247893497
0.552414894	0.901856601	0.427567601
0.796162009	0.954407573	0.015359100
0.801188111	0.224413797	0.204465300
0.817156911	0.470851004	0.376600593
0.828279793	0.258218110	0.076367803
0.796802282	0.499630004	0.247437105
0.825186491	0.760251999	0.427433014
0.577546895	0.212238699	0.014761900
0.533841014	0.446794599	0.195568293
0.555499494	0.692392170	0.376767099
0.552767515	0.151497394	0.148215801
0.533602417	0.409575611	0.332103193
0.540808201	0.674676895	0.498790205
0.835118592	0.757811010	0.074706502
0.821304321	0.007196700	0.247510403
0.824196517	0.258328289	0.424864292
0.819442987	0.018131699	0.147448793
0.800590873	0.268635511	0.323940814
0.844322205	0.490442395	0.497465700
0.550356388	0.903333008	0.077249102
0.544924617	0.137048900	0.250255287
0.554810703	0.400854409	0.430094600
0.579854906	0.713572204	0.014323700
0.554889977	0.938367784	0.198573604
0.551100314	0.192838699	0.376091987
0.686945021	0.332067788	0.036458299
0.677354991	0.580859482	0.198211595
0.688481510	0.830785811	0.377572596
0.690648973	0.832398772	0.036177501
0.683217525	0.081230797	0.199479297
0.681454778	0.333407789	0.377414703
0.209796801	0.300114393	0.254770190
0.179120094	0.591612577	0.198762596
0.184066504	0.333469599	0.043385100
0.188154399	0.833482921	0.380777091
0.186472505	0.345111996	0.390047699
0.183005899	0.101110898	0.200731203
0.191159800	0.835926175	0.044949502
0.686079621	0.582469523	0.074763298
0.682107627	0.829298079	0.260716796

0.686124086	0.081408098	0.438229889
0.691543102	0.333750904	0.145449102
0.686957598	0.579791784	0.318333298
0.691073596	0.832687080	0.492782801
0.681186199	0.082926102	0.075469702
0.614865303	0.330927104	0.260390401
0.687147915	0.581172824	0.439451009
0.705708802	0.830447614	0.144757599
0.694284678	0.081869103	0.318620503
0.701792598	0.328306496	0.492589593

Ca-7_{H2O}-LDH
1.0

7.8200001717	0.0000000000	0.0000000000
-0.0000031182	11.7086601257	0.0000000000
-0.0000037977	-0.0000037977	28.5200004578

H	O	Al	Cl	Ca
38	37	6	6	12

Direct

0.938167989	0.526262999	0.153220996
0.946169972	0.771997988	0.333389997
0.963334978	0.998956025	0.499020010
0.918923020	0.442140996	0.009383000
0.942698002	0.696134984	0.202885002
0.944196999	0.954590976	0.378381014
0.431295007	0.654464006	0.151026994
0.427323997	0.893975973	0.335420996
0.419220001	0.165875003	0.502696991
0.419916987	0.389272004	0.071102999
0.420320004	0.643978000	0.242723003
0.428793013	0.888957024	0.421362013
0.916667998	0.948119998	0.008182000
0.922150016	0.212687999	0.207819000
0.941533983	0.455538005	0.379446000
0.952422023	0.274103999	0.071117997
0.920323014	0.496594012	0.242311001
0.948234975	0.773191988	0.420911014
0.456746012	0.222194999	0.007753000
0.410292000	0.467770994	0.193054006
0.432628006	0.708468020	0.378219992
0.423963010	0.148999006	0.153026000
0.417080998	0.411518008	0.342516005
0.421990007	0.663155019	0.505195022

0.960547984	0.773562014	0.069040999
0.939382017	0.017849000	0.241999000
0.945882976	0.269443989	0.417317986
0.939321995	0.033893000	0.153331995
0.921483994	0.284702986	0.325168997
0.966345012	0.505548000	0.502151012
0.426173002	0.885451019	0.072727002
0.416200995	0.124164999	0.244720995
0.430651009	0.388327003	0.426297009
0.459138006	0.723675013	0.008028000
0.427089006	0.950119019	0.195976004
0.426384985	0.206863001	0.377593011
0.235567003	0.324315995	0.295118988
0.230088994	0.267628014	0.244682997
0.815164983	0.515303016	0.147244006
0.823103011	0.762166023	0.327690989
0.839743018	0.994075000	0.497267008
0.796609998	0.451950014	0.015462000
0.820029020	0.717001975	0.203024998
0.820950985	0.970995009	0.376509994
0.555803001	0.657796979	0.146380007
0.550036013	0.902417004	0.328615010
0.541297972	0.174098000	0.498066992
0.544372976	0.405203015	0.076358996
0.543909013	0.651867986	0.248101994
0.551401973	0.902099013	0.427623004
0.795136988	0.954985023	0.015629999
0.798269987	0.223740995	0.204760000
0.818520010	0.471500993	0.377766997
0.827890992	0.258653998	0.076862000
0.796809018	0.500181973	0.247390002
0.825523019	0.761151016	0.427406013
0.578029990	0.212316006	0.015062000
0.532325983	0.448211998	0.195078999
0.555975020	0.692565978	0.376922011
0.547867000	0.151813999	0.147922993
0.538174987	0.413776010	0.333467007
0.542967975	0.674395978	0.499579996
0.835960984	0.758009017	0.074956000
0.816582978	0.006802000	0.247397006
0.824508011	0.259054005	0.425024986
0.817420006	0.019359000	0.147772998
0.798904002	0.268833011	0.324378014

0.846625984	0.488972008	0.496809006
0.550638974	0.902889013	0.077579997
0.538779974	0.137584001	0.249752000
0.553357005	0.400810003	0.430687994
0.581071973	0.713482976	0.014459000
0.550424993	0.938641012	0.198137999
0.550103009	0.193804994	0.376540005
0.287178993	0.327125013	0.263498008
0.686951995	0.332215995	0.036787000
0.676778972	0.581974030	0.198043004
0.687933028	0.831514001	0.377478004
0.690810978	0.832502007	0.036476001
0.678050995	0.080999002	0.199267998
0.681532979	0.334208995	0.377620012
0.178672001	0.595080972	0.193856999
0.184508994	0.332345009	0.042146001
0.187801003	0.833301008	0.381677985
0.178790003	0.331597000	0.373414010
0.172382995	0.107257001	0.202500999
0.192913994	0.833688021	0.043116000
0.685209990	0.582868993	0.075346999
0.677928984	0.829986989	0.260443002
0.684629977	0.081295997	0.438089997
0.689734995	0.333770007	0.145884007
0.691213012	0.581596017	0.318190008
0.691056013	0.833468974	0.492599010
0.680094004	0.083113998	0.076041996
0.605571985	0.332612008	0.260625005
0.687623024	0.582295001	0.439740986
0.707006991	0.831318974	0.145153001
0.690280020	0.082079999	0.317948997
0.703473985	0.327805996	0.493153989

Ca-7$_{OH}$-LDH

1.0

7.8200001717	0.0000000000	0.0000000000
-0.0000031182	11.7086601257	0.0000000000
-0.0000037977	-0.0000037977	28.5200004578

H	O	Al	Cl	Ca
37	37	6	6	12

Direct

0.938507497	0.522522509	0.153326005
0.946311295	0.768246710	0.333917707

0.963250816	0.000645500	0.499616504
0.920218110	0.441450894	0.009757800
0.943055987	0.694079578	0.202998102
0.945879400	0.952571809	0.378889114
0.430591196	0.651376903	0.150192007
0.429369688	0.894002080	0.335279107
0.420043409	0.168361902	0.500999629
0.419792712	0.389691114	0.070968397
0.420972914	0.642760813	0.243136793
0.429471791	0.889636278	0.421221107
0.918959796	0.945805907	0.008624000
0.922849715	0.216346696	0.209877402
0.939629078	0.453883201	0.379239798
0.952932477	0.275465608	0.071125202
0.918516099	0.497727603	0.243490905
0.948297679	0.772916317	0.421160489
0.457641900	0.221369505	0.007196800
0.412212014	0.467553914	0.192058697
0.432051390	0.708129823	0.378680706
0.428239793	0.150423706	0.153780594
0.410064787	0.404920995	0.341875702
0.419599295	0.662922978	0.502786815
0.959123611	0.774585426	0.069234602
0.945190012	0.019925799	0.243406907
0.946202576	0.273286194	0.418282807
0.941631377	0.031169901	0.151538596
0.919891477	0.286074787	0.322421700
0.966545403	0.501613081	0.500610709
0.424655706	0.886943221	0.071853600
0.420568109	0.129622906	0.246242002
0.429729015	0.390343904	0.425091803
0.457400292	0.723272204	0.008300900
0.430880994	0.951264799	0.198548600
0.426447690	0.206827804	0.378669709
0.215403304	0.348714113	0.231558606
0.815411508	0.515383720	0.146740705
0.823335707	0.759669304	0.327748209
0.840218008	0.992643416	0.497169286
0.797353387	0.451660603	0.015143400
0.821672201	0.716287076	0.202181205
0.822830975	0.969233811	0.376571387
0.554631889	0.656537712	0.146171898
0.552087605	0.901525617	0.328366607

0.543500006	0.172233298	0.498429507
0.544650614	0.405301809	0.076044597
0.544402480	0.653612375	0.247347295
0.552174389	0.902050018	0.427474409
0.796895504	0.954014897	0.015283900
0.799553096	0.223985493	0.204578295
0.816691816	0.470551610	0.376360297
0.828277886	0.258614808	0.076590799
0.794217825	0.499926388	0.247533202
0.825635672	0.760545373	0.427462190
0.578545213	0.211993605	0.014927700
0.534461379	0.448241502	0.194673106
0.555263102	0.692245603	0.376891404
0.551981091	0.150552496	0.148163900
0.530886412	0.409766495	0.332347810
0.541483223	0.674063027	0.498768896
0.834303498	0.758076429	0.074885502
0.822576225	0.006771900	0.247315705
0.823704898	0.258599788	0.424619406
0.819284081	0.017466400	0.147577703
0.798029900	0.268598408	0.323677301
0.844227493	0.490676999	0.497483402
0.549551785	0.903814375	0.076824702
0.544736385	0.133514896	0.250731796
0.553713977	0.401325196	0.430027902
0.579590201	0.713291705	0.014434500
0.553865612	0.938086808	0.198629498
0.549726009	0.192716405	0.376354903
0.282404393	0.300307214	0.252957106
0.687085271	0.332165509	0.036595698
0.677742600	0.580692828	0.198090896
0.688545585	0.830650926	0.377567708
0.689990222	0.832486689	0.036143899
0.682198405	0.080941297	0.199729204
0.680027723	0.333310902	0.377236307
0.172253594	0.567113876	0.200948194
0.182784796	0.336227089	0.043883700
0.188377097	0.833037615	0.381150186
0.184984893	0.344170004	0.390012801
0.182990998	0.128962800	0.203785300
0.190303698	0.835520327	0.045006499
0.686936796	0.582260072	0.074600004
0.683405697	0.829726398	0.260987699

0.686834216	0.081442401	0.437968612
0.690932989	0.333356798	0.145606399
0.684972227	0.579124987	0.318297714
0.690466881	0.832843482	0.492579997
0.682043612	0.082897402	0.075478099
0.601153493	0.329382509	0.260025114
0.688155293	0.581207395	0.439124286
0.702443004	0.830601692	0.144643903
0.692842185	0.081850499	0.318791211
0.701189399	0.328584611	0.492213100

Reference

1. G. Kresse and J. Hafner, Ab initio molecular dynamics for liquid metals, *Phys. Rev. B*, 1993, **47**, 558-561.
2. G. Kresse and J. Hafner, Ab initio molecular-dynamics simulation of the liquid-metal--amorphous-semiconductor transition in germanium, *Phys. Rev. B*, 1994, **49**, 14251-14269.
3. G. Kresse and J. Furthmüller, Efficient iterative schemes for ab initio total-energy calculations using a plane-wave basis set, *Phys. Rev. B*, 1996, **54**, 11169-11186.
4. P. E. Blöchl, Projector augmented-wave method, *Phys. Rev. B*, 1994, **50**, 17953-17979.
5. G. Kresse and D. Joubert, From ultrasoft pseudopotentials to the projector augmented-wave method, *Phys. Rev. B*, 1999, **59**, 1758-1775.
6. J. P. Perdew, K. Burke and M. Ernzerhof, Generalized Gradient Approximation Made Simple, *Phys. Rev. Lett.*, 1996, **77**, 3865-3868.
7. H. J. Monkhorst and J. D. Pack, Special points for Brillouin-zone integrations, *Phys. Rev. B*, 1976, **13**, 5188-5192.
8. D. J. Chadi, Special points for Brillouin-zone integrations, *Phys. Rev. B*, 1977, **16**, 1746-1747.
9. H. Yan, X.-J. Zhao, Y.-Q. Zhu, M. Wei, D. G. Evans and X. Duan, in *The Periodic Table II: Catalytic, Materials, Biological and Medical Applications*, ed. D. M. P. Mingos, Springer International Publishing, Cham, 2019, DOI: 10.1007/430_2019_47, pp. 89-120.
10. X. Kong, R. Ge, T. Liu, S. Xu, P. Hao, X. Zhao, Z. Li, X. Lei and H. Duan, Super-stable mineralization of cadmium by calcium-aluminum layered double hydroxide and its large-scale application in agriculture soil remediation, *Chem. Eng. J.*, 2021, **407**.
11. C. Hartwigsen, S. Goedecker and J. Hutter, Relativistic separable dual-space Gaussian pseudopotentials from H to Rn, *Phys. Rev. B*, 1998, **58**, 3641-3662.
12. S. Goedecker, M. Teter and J. Hutter, Separable dual-space Gaussian pseudopotentials, *Phys. Rev. B*, 1996, **54**, 1703-1710.
13. J. VandeVondele and J. Hutter, Gaussian basis sets for accurate calculations on molecular systems in gas and condensed phases, *J. Chem. Phys.*, 2007, **127**, 114105.
14. G. J. Martyna, M. L. Klein and M. Tuckerman, Nosé–Hoover chains: The canonical ensemble via continuous dynamics, *J. Chem. Phys.*, 1992, **97**, 2635-2643.
15. W. G. Hoover, Canonical dynamics: Equilibrium phase-space distributions, *Phys. Rev. A*, 1985, **31**, 1695-1697.
16. M. Brehm, M. Thomas, S. Gehrke and B. Kirchner, TRAVIS—A free analyzer for trajectories from molecular simulation, *The Journal of Chemical Physics*, 2020, **152**.
17. F. Jalilehvand, D. Spångberg, P. Lindqvist-Reis, K. Hermansson, I. Persson and M. Sandström, Hydration of the Calcium Ion. An EXAFS, Large-Angle X-ray Scattering, and Molecular Dynamics Simulation Study, *J. Am. Chem. Soc.*, 2001, **123**, 431-441.
18. Y. Li, H. Pan, Q. Liu, X. Ming and Z. Li, Ab initio mechanism revealing for tricalcium silicate dissolution, *Nat. Commun.*, 2022, **13**, 1253.
19. C. Lefebvre, G. Rubez, H. Khartabil, J.-C. Boisson, J. Contreras-García and E. Hénon, Accurately extracting the signature of intermolecular interactions present in the NCI plot of the reduced density gradient versus electron density, *Phys. Chem. Chem. Phys.*, 2017, **19**, 17928-17936.
20. T. Lu and F. Chen, Multiwfn: A multifunctional wavefunction analyzer, *J. Comput. Chem.*, 2012, **33**, 580-592.

21. T. Lu and Q. Chen, Independent gradient model based on Hirshfeld partition: A new method for visual study of interactions in chemical systems, *J. Comput. Chem.*, 2022, **43**, 539-555.
22. T. Lu and Q. Chen, in *Reference Module in Chemistry, Molecular Sciences and Chemical Engineering*, Elsevier, 2023, DOI: https://doi.org/10.1016/B978-0-12-821978-2.00076-3.
23. W. Xiong, J. Wang, X. Kong, G. I. N. Waterhouse, H. Liu, Y. Wang, S. Liu, Y. Wang, S. Li, Y. Zhao and H. Duan, Efficient and Superstable Mineralization of Toxic Cd2+ Ions through Defect Engineering in Layered Double Hydroxide Nanosheets, *J. Phys. Chem. C*, 2023, **127**, 8759-8769.
24. W. Xiong, M. Yang, J. Wang, H. Wang, P. Zhao, Z. Li, B. Liu, X. Kong, H. Duan and Y. Zhao, Removal, recycle and reutilization of multiple heavy metal ions from electroplating wastewater using super-stable mineralizer Ca-based layered double hydroxides, *Chem. Eng. Sci.*, 2023, **279**.
